# Antimicrobial and antibiofilm effects of crude and microencapsulated guava leaf extracts against *Enterococcus faecalis* and *Staphylococcus epidermidis*

**DOI:** 10.3389/frabi.2025.1615787

**Published:** 2025-12-03

**Authors:** Daniela Gutierrez-Montiel, Alma L. Guerrero-Barrera, Adriana C. Moreno-Flores, Flor Y. Ramírez-Castillo, Norma A. Chávez-Vela, Guillermo Cristian Guadalupe Martínez-Ávila, Roberto Rico-Martínez, Matheus de O. Costa, Fabiola Galindo-Guerrero, Francisco J. Avelar-Gonzalez, Ingrid G. Ornelas-Garcia, Omar Franco-Ramírez

**Affiliations:** 1 Laboratorio de Biología Celular y Tisular, Centro de Ciencias Básicas, Departamento de Morfología, Universidad Autónoma de Aguascalientes, Aguascalientes, Mexico; 2 Laboratorio de Biotecnología, Centro de Ciencias Básicas, Departamento Ingeniería Bioquímica, Universidad Autónoma de Aguascalientes, Aguascalientes, Mexico; 3 Laboratorio de Química y Bioquímica, Facultad de Agronomía, Universidad Autónoma de Nuevo León, General Escobedo, Mexico; 4 Laboratorio de Toxicología Acuática, Departamentos de Química y Biología, Universidad Autónoma de Aguascalientes, Aguascalientes, Mexico; 5 Large Animal Clinical Sciences, Western College of Veterinary Medicine, University of Saskatchewan, Saskatoon, SK, Canada; 6 Population Clinical Sciences, Faculty of Veterinary Medicine, Utrecht University, Utrecht, Netherlands; 7 Laboratorio de Estudios Ambientales, Centro de Ciencias Básicas, Departamento de Fisiología y Farmacología, Universidad Autónoma de Aguascalientes, Aguascalientes, Mexico; 8 Department of Data Science, Escuela de Ciencia y Tecnología de la Universidad de Aguascalientes, Aguascalientes, Mexico

**Keywords:** guava leaf, antimicrobial, antibiofilm, plant extracts, antiadhesion effects, *Psidium guajava* L.

## Abstract

**Background:**

*Staphylococcus epidermidis* and *Enterococcus faecalis* are nosocomial microorganisms that have gained attention in recent times due to the increasing reports of antimicrobial-resistant strains, which are leading to infections that are progressively harder to eradicate. One of the most important resistance mechanisms employed by these two bacteria is biofilm formation, which provide them with physical and chemical protection from antimicrobial agents.

**Methods:**

This study assessed the antimicrobial activity of crude and microencapsulated extracts of *Psidium guajava* L., an agro-industrial waste product widely available in guava-producing countries, using the microdilution technique. Additionally, anti-adhesion activity was analyzed in microplates and by confocal laser scanning microscopy (CLSM).

**Results:**

Guava leaf extract reduced the growth of all three bacterial strains evaluated. For *Staphylococcus epidermidis* (ATCC 12228), the minimum inhibitory concentrations (MICs) were 25 mg/ml for the crude extract and 0.625 mg/ml for the microencapsulated form. In contrast, for *Enterococcus faecalis* (ATCC 29212 and a vaginal clinical isolate), MIC values were greater than 50 mg/ml and 5 mg/ml, respectively. Furthermore, both extracts exhibited anti-biofilm activity by reducing bacterial adhesion.

**Conclusion:**

microencapsulation allowed a reduction in the extract concentration and guava leaf extract shows potential as an antimicrobial agent for future application.

## 1 Introduction

Currently, it is estimated that antimicrobial resistance (AMR) causes approximately 700,000 deaths each year, a number that could rise to 10 million by 2050 if effective measures are not implemented to curb its spread (Rehman, 2023; Ahmed et al., 2024). As a result, AMR, often referred to as the “silent pandemic”, is considered one of the most significant global health challenges of the 21st century (Coque et al., 2023). Biofilm formation is a main strategy to resist antimicrobial exposure (Uruén et al., 2020). Biofilms are communities of microorganisms embedded in a extracellular matrix composed by extracellular polymeric substances that allow them to adhere to any surface, including metals, plastics, particles, and biotic surfaces (Mgomi et al., 2023).

In recent years, *Enterococcus* spp. have attracted significant attention among the multidrug-resistant (MDR) bacteria responsible for nosocomial infections, particularly due to their ability to cause urinary tract infections (UTIs), bacteremia, and infective endocarditis (Boccella et al., 2021). *E. faecalis* is a commensal bacterium found in the human microbiota; however, it is an opportunistic pathogen (Mechmechani et al., 2022) and one of the most prevalent species of the *Enterococcus* genus isolated from clinical samples. Moreover, it is one of the main pathogens on patients with UTIs worldwide (Ma et al., 2021). Besides, *E. faecalis* can exhibit resistance to several commonly used antibiotics, including aminoglycosides and macrolides (Wen et al., 2020). A major resistance mechanisms of *E. faecalis* is its ability to form biofilms, which has also been associated with persistent host inflammation (Zeng et al., 2021). Similarly, *S. epidermidis* a commensal bacterium on human skin, has been reported to form biofilms on different medical devices, such as intravenous catheters, prosthetic joints and heart valves, facilitating its entry into the body and contributing to infections (Foster, 2020). Therefore, it is recognized as an important opportunistic pathogen, with biofilm formation being a key factor in its ability to establish infections (Guo et al., 2019). *S. epidermidis* strains usually exhibit resistance to several types of antibiotics, such as tetracyclines, aminoglycosides, cephalosporins, fluoroquinolones, penicillins, and macrolides, becoming a serious problem in hospital settings (Chabi and Momtaz, 2019).

In general, biofilms reduce the possibility of eradicating infections and contribute to relapses even after adequate traditional treatment. Furthermore, they not only increase severity of symptoms but also enhance mortality rates (Zhang et al., 2020). Considering the stages of biofilm formation, antibiofilm strategies can be target several mechanisms, including the inhibition of microbial adhesion to surfaces, interference with signal molecules involved in biofilm development (*quorom-sensing*), and the disruption of the biofilm matrix (Andrade et al., 2020).

In this context, phytochemicals have emerged as promising alternatives given their great structural diversity and multiple modes of action, which make them effective even against multidrug-resistant (MDR) strains (Álvarez-Martínez et al., 2021). *Psidium guajava* L., a shrub native to America and belonging to the Myrtacea family, has been extensively investigated for its leaves, which are rich in phenolic compounds that confer antioxidant, anti-inflammatory, and antimicrobial properties. In recent years, it has also been reported that this plant material may exhibit antibiofilm activity. In clinical settings, bacteria predominantly persist within biofilms, which provide structural protection and markedly enhance resistance to antimicrobial agents. In this context, the antibiofilm potential of guava leaf extract against clinically relevant pathogens such as *Staphylococcus epidermidis* and *Enterococcus faecalis* remains poorly investigated, the limited studies published in this field have mainly focused on its effects against *Staphylococcus aureus* (Sakarikou et al., 2020; Gutierrez-Montiel et al., 2023). Therefore, the present study aims to evaluate the antimicrobial and antibiofilm activity of methanolic guava leaf extract against *E. faecalis* and *S. epidermidis*. Furthermore, the influence of microencapsulating purified polyphenols in β-cyclodextrins on its antimicrobial activity was also assessed. To our knowledge, such an approach has not yet been examined with respect to its impact on bacterial adhesion and biofilm formation in these clinically relevant strains.

## 2 Materials and methods

### 2.1 Collection of plant material

Green and healthy guava leaves were manually and randomly collected from different pesticide-free specimens in Aguascalientes, Mexico. The leaves were then washed with distilled water to remove dust residues and other contaminants and dried at 40 °C for 72 hours (Shabbir et al., 2020). Finally, the leaves were pulverized using an electric grinder, and the resulting powder was stored in an airtight container, protected from light at room temperature (Azizan et al., 2020).

### 2.2 Extraction of phytochemicals

Phytochemical extraction was carried out as previously reported by Gutierrez-Montiel, et al (Gutierrez Montiel et al., 2023; Gutierrez-Montiel et al., 2024). Briefly, plant extract was obtained using a Soxhlet apparatus with a solid-liquid ratio of 1:20 (5 g of plant material per 100 ml of solvent), using methanol as the extraction solvent for seven siphons. The resulting extract was diluted with distilled water to achieve an 80% methanol solution, then placed in an oven at 50°C to evaporate the solvent, resulting in an aqueous fraction with a concentration of 100 mg/ml (stock solution) (Castro-López et al., 2016; Castro-López et al., 2019; Gutierrez-Montiel et al., 2024). Finally, the extract was filtered with 0.2 µm membranes (Díaz-de-Cerio et al., 2016) and stored in the dark at 4°C until further use (Biswas et al., 2013). Throughout the manuscript, this extract is referred to as the crude extract (GLE).

### 2.3 Phytochemical tests

Qualitative tests were carried out for the detection of saponins, phenols, terpenes, and flavonoids in the crude guava leaf extract (Tanaka et al., 1992; Biswas et al., 2013; Le Thi et al., 2021). The presence of saponins was confirmed by the formation of foam after shaking the extract in a test tube. On the other hand, for the phenols detection, the extract was mixed with 2 ml of 2% ${\text{FeCl}}_{3}$, a dark blue coloration indicated a positive reaction. Similarly, to confirm the presence of terpenes, the extract was first mixed with 2 ml of chloroform and then with 2 ml of HCl and mixed gently, a reddish-brown interface indicated a positive result. And finally, 2 ml of 10% NaOH was added to the extract to confirm the presence of flavonoids, an intense yellow color indicated a positive reaction. Details regarding the characterization of the guava leaf extract used in this study are provided in a previous publication (Gutierrez Montiel et al., 2023).

### 2.4 Microencapsulation by spray drying

Phenolic compounds from guava leaves were purified using Amberlite XAD-16 resin. The extract was loaded onto a column, washed with distilled water to remove sugars and impurities, and then eluted with absolute ethanol. The solvent was evaporated at 50 °C for 24 hours to obtain crystalline polyphenols (Castro-López et al., 2019; Gutierrez-Montiel et al., 2024). Purified guava leaf polyphenols (GLEP) were dissolved in 100 ml of a 15% ethanol–water solution at a concentration of 15 mg/ml. The mixture was heated at 40 °C for 10 minutes, mixed with β-cyclodextrin (Sigma-Aldrich, St. Louis, MO, USA) at a 1:2 ratio and stirred at 50 °C for 30 minutes to ensure proper homogenization. The resulting solution was filtered through a 0.25 µm membrane. Finally, encapsulation was performed in a pilot scale spray dryer (NIRO-Productionmirror^®^, Alemania) with an inlet air temperature of 180°C, outlet temperature of 90°C, feed rate of 2 ml/min, and atomization speed of 26,350 rpm. Resulting powder was stored in Eppendorf tubes, protected from light at room temperature. Throughout the manuscript, this extract is referred to as the microencapsulated extract (GLEM) (Delgadillo-Aguirre, 2019; Castro-López et al., 2021; Dobroslavić et al., 2023). This technique provides an effective means to protect and enhance the stability of phytochemicals by incorporating them into cyclodextrins, cyclic oligosaccharides with a hydrophobic internal cavity and a hydrophilic external surface. Owing to their high biocompatibility and approval by the U.S. Food and Drug Administration (FDA), beta-cyclodextrins are considered a safe and suitable vehicle for delivering bioactive compounds (Escobar-Avello et al., 2021; Wüpper et al., 2021; Kuzmanović Nedeljković et al., 2023).

### 2.5 Determination of the properties of the microencapsulated extract

#### 2.5.1 Scanning electron microscopy

The crude, purified, and microencapsulated guava leaf extracts were analyzed by scanning electron microscopy (SEM) using a JSM-5900LV system (JEOL Ltd., Tokyo, Japan). The microscope was operated at 20 kV accelerating voltage. The samples were coated with a 100 Å-thick layer of gold using a Denton Vacuum DESK II sputter coater (Denton Vacuum, USA).

#### 2.5.2 Encapsulation efficiency

The amount of guava leaf extract encapsulated within β-cyclodextrins was quantified spectrophotometrically. A UV-Vis spectral scan of the purified extract (the same used for microencapsulation) was carried out using a HACH DR/4000U UV–Visible spectrophotometer (Loveland, CO, USA). The sample was scanned over the wavelength range of 200–700 nm to determine the maximum absorption wavelength, which was identified at 247 nm. Subsequently, a calibration curve was constructed by measuring the absorbance of a series of known concentrations of the purified extract at this wavelength. For the analysis of the encapsulated sample, 5 mg of the microencapsulated powder were dissolved in 5 ml of absolute ethanol, and the absorbance was measured at 247 nm using absolute ethanol as the blank. The concentration of GLEP in the solution was determined using the calibration curve (Wu et al., 2022b; Chang et al., 2023). Encapsulation efficiency (EE) was calculated using the following equation (Wu et al., 2022a).


$$EE(\%)=\frac{\text{total amount of GLEP encapsulated}}{\text{initial total GLEP added in solution}}$$

#### 2.4.3 Moisture content

The moisture content of the powder was determined by measuring the weight loss after placing 100 mg of the encapsulated extract in a porcelain crucible and drying it in an oven at 105 °C until a constant weight was achieved (approx. 4 hours) (Muñoz-Molina et al., 2025).

#### 2.4.4 Solubility

Solubility was determined based on the protocol described by Hussain (Hussain et al., 2018), with minor modifications. Briefly, 150 mg of the microencapsulated powder was added to 30 ml of distilled water and stirred using a magnetic stirrer for 20 minutes. The solution was then transferred to 50 ml Falcon tube and centrifuged at 3000 rpm for 10 minutes. Subsequently, a 10 ml aliquot of the supernatant was transferred to pre-weighed Petri dishes and dried in an oven at 105 °C for 4 hours, until a constant weight was reached. Solubility was expressed as the percentage of soluble solids relative to the initial mass of the powder.

### 2.6 Microorganisms and culture media

Two different strains of *Enterococcus faecalis* were evaluated: a clinical isolate from a vaginal exudate, obtained from the Microbiology Laboratory of the Autonomous University of Aguascalientes, and the strain ATCC 29212 (American Type Culture Collection). Besides, *Staphylococcus epidermidis* ATCC 12228 was included in this study. All bacteria were cultured in brain heart infusion (BHI) broth and agar medium (BD Bioxon, Le Pont de Claix, France). The clinical bacteria was initially donated. However, to confirm the isolate as *E. faecalis*, the clinical bacteria was growth in BBL™ Enterococcosel™ Agar (BD Bioxon, Le Pont de Claix, France). Black and small colonies were indicative of *Enterococcus* sp. After that, colonies were growth in salt and mannitol agar (Bioxon, Mexico), and catalase test was made (catalase negative). All the isolates were kept in BHI plus glycerol 20% (v/v) to – 80 °C until it uses. For DNA extraction, bacteria were cultured on BHI agar for 24 h at 37 ° C. DNA isolation was performed as described by Sambrook & Russell (Sambrook and Russel, 2001). Additionally, confirmation was PCR was carried out with the following primers: Ef-pepA-F 5’-CCAAGCCACGTAATGCTTGT-3’, and Ef-pepA-R 5’-TAGCAGCTCACATGGACGAA-3’ given an amplicon of 849 pb (designed by this study). The PCR cycle included initial denaturation at 95 °C for 5 min; then 30 cycles of denaturation at 95 °C for 1 minute, annealing at 53 °C for 30 seconds, and elongation at 72 °C for 1 minute; and final elongation at 72 °C for 10 minutes. *E. faecalis* ATCC 29212 was used as positive control and water as negative control.

Additionally, disk diffusion method was used to test the antibiotic susceptibility patterns of the *E. faecalis* isolate as previously described by CLSI (Clinical and Laboratory Standards Institute (CLSI), 2023). *E. faecalis* ATCC 29212 was used as control. Antibiotic tested were: ampicillin (AM, 10 µg), vancomycin (VA, 30 µg), erythromycin (E, 15 µg), tetracycline (TE, 30 µg), doxycycline (DC, 30 µg), ciprofloxacin (CPF, 5 µg), clindamycin (CLM, 2 µg), cephalothin (CF, 30 µg), cefotaxime (CFX, 2 µg), penicillin (10 units), and trimethoprim-sulfamethoxazole (1.25/23.75 µg).

### 2.7 Determination of the minimum inhibitory concentration and minimum bactericidal concentration

The minimum inhibitory concentration (MIC) and the minimum bactericidal concentration (MBC) were determined in 96-well microplates (Costar 3370, Corning, NY, USA) by performing two-fold serial dilutions of the guava leaf extract, with concentrations ranging from 6.25 to 50 mg/ml for the crude extract and 0.3125 to 5 mg/ml for the microencapsulated. Gentamicin at 5 µg/ml and chlorhexidine at 0.6% were used as positive controls. The bacterial concentration was adjusted to the 0.5 McFarland’s standard. All assays were performed in triplicate. To prepare the 96-well plates, 50 µl of the standardized inoculum was dispensed into each well, followed by the addition of 50 µl of the extract at different concentrations. The final volume in each well was 100 µl. Growth controls and blanks (containing medium and extract but no inoculum, at each tested concentration) were included for each strain. The microplate was incubated at 37 °C for 24 h and subsequently agar plates were inoculated (in triplicate) to count the CFU. Optical density (595 nm) was measured using a spectrophotometer (Benchmark plus Microplate Reader, BIO-RAD, Hercules, CA). The MIC was defined as the lowest concentration of the extract required to inhibit bacterial growth (absence of turbidity); while MBC was defined as the lowest concentration that killed 99% of the bacteria, showing no growth on agar plates (Mogana et al., 2020; Saeloh and Visutthi, 2021; Gutierrez-Montiel et al., 2024; Surana et al., 2024).

### 2.8 Effect of guava leaf extract on bacterial adhesion

The effect of *P. guajava* L. extract on biofilm formation was evaluated using 96-well microplates (Costar 3370, Corning, NY, USA). Briefly, overnight cultures were diluted 1:100 in BHI broths with 0.8% glycerol (Ramírez-Castillo et al., 2018; Ramírez Castillo et al., 2023), and 100 µl of the diluted culture were aliquoted in triplicate into a sterile microtiter plate. Then, 100 µl of guava leaf extract at different concentrations (6.25 to 50 mg/ml for the crude extract and 0.3125 mg/ml to 2.5 mg/ml for the microencapsulated) were added to each well. Distilled water was used as the negative control. Gentamicin (Thermo Fisher Scientific, Gibco™) at 5 µg/ml and a commercial mouthwash originally containing 0.12 % chlorhexidine (Bexident^®^, ISDIN, Spain), diluted to a final concentration of 0.06 %, were used as positive controls. The culture medium supplemented with either water or the extract (at each concentration tested) served as the blank. The plate was incubated at 37 °C for 24 h, and then it was stained with crystal violet (Sandasi et al., 2010; Adnan et al., 2020) as follows: first, the culture medium with planktonic cells was discarded using a multichannel pipette, and the plate was washed twice with distilled water before being air-dried at 37° C for approximately 30 min. Finally, 150 µl of crystal violet were added and left for 2 minutes before removed by immersing the plate in a tub of water. The plate was able to dry completely before adding 150 µl of 30% acetic acid. The optical density (OD) was measured at 595 nm using a Benchmark plus Microplate Reader (BIO-RAD, Hercules, CA) (Ramírez-Castillo et al., 2018). The inhibition percentage was estimated as follows (Sandasi et al., 2010):


$$Percentage\;inhibition=\;\frac{OD_{Negative\;control\;}-\;OD_{Experimental\;\;}\;}{OD_{Negative\;control\;}}\times 100$$

### 2.9 Confocal laser scanning microscopy analysis

To further confirm the results of bacterial adhesion from the crystal violet assay, the effect of guava leaf extract on biofilm formation was analyzed by confocal laser scanning microscopy (CLSM). For biofilm formation, a sterile slide was poured in a Petri dish along with BHI nutrient medium containing 0.8% glycerol, a 1:10 dilution of a 24-h bacteria culture, and sub-inhibitory concentrations of guava leaf extract. For *S. epidermidis*, a concentration of 6.25 mg/ml was used, while 25 mg/ml was selected for the two strains of *E. faecalis*. The plates were incubated for 24 hours and then stained according to the supplier’s instructions. Briefly, the slides were fixed by exposure to 80% ethanol for 15 min and dried to room temperature. Then, the slides were treated with 200 µl of the commercial dye FilmTracer FM 1-43 (Invitrogen, Eugene, OR) to stain the extracellular matrix. The samples were incubated for 30 min at room temperature, protected from light, and rinsed gently with distilled water to remove excess dye. Finally, the coverslips were mounted with ProLong Gold anti-fade reagent (Invitrogen, Eugene, OR) and observed using a confocal microscope (CLSM; LMS 700 ZEISS; Carl Ziess Microscopy, Jena, Germany) with an EC PlnN 40X 1.3 NA Oil DICIII objective. Images were acquired using Zen Black 2012 (black edition) software (ZEISS).

To assess the effect of guava leaf extract on the extracellular polymeric substances (EPS) of the biofilm, a triple fluorescent staining protocol was performed. Biofilms were prepared as previously described and stained with Wheat Germ Agglutinin (WGA, Oregon Green 488; Molecular Probes) to detect N-acetyl-D-glucosamine (PNAG) and N-acetyl-neuraminic acid residues (NANA), SYPRO^®^ Ruby biofilm matrix stain (Molecular Probes) to label proteins, and DAPI (4′,6-diamidino-2-phenylindole dihydrochloride; Thermo Fisher Scientific, cat. no. D1306) to visualize extracellular DNA (eDNA), according to the manufacturers’ instructions. Stained samples were examined using confocal laser scanning microscopy (CLSM, LSM 700, ZEISS, Jena, Germany), and representative images were acquired with Zen Black 2012 software (ZEISS).

### 2.10 Acute toxicity assay using *Lecane papuana* and *Paramecium caudatum*

As a preliminary approach to assess the potential toxicity of *Psidium guajava* L., the acute effects of its crude leaf extract were evaluated in the freshwater rotifer *Lecane papuana* and the ciliate *Paramecium caudatum* following the protocol of Flesch et al (Flesch et al., 2019). *L. papuana* was cultured in EPA medium supplemented with *Nannochloris oculata*, while *P. caudatum* was maintained in wheat infusion. Toxicity tests were conducted by exposing 10 individuals to increasing concentrations of the crude extract for 24 hours: 0, 1, 3, 6, 8, and 15 mg/ml for *L. papuana* and 0, 0.5, 1, 2, 3, 4, and 5 mg/ml for *P. caudatum*. After exposure, live and dead organisms were counted under a stereomicroscope. The results are expressed as LC_50_, the concentration at which 50% mortality is observed compared to the control group; LOEC (Lowest Observed Effect Concentration) and NOEC (No Observed Effect Concentration).

### 2.11 Cell viability and cytotoxicity assays

#### 2.11.1 Cell culture

Green monkey kidney epithelial cells (Vero-76) were cultured in DMEM/F12 medium (1:1) (Gibco, USA) supplemented with 10% fetal bovine serum (FBS; Gibco, USA) and 1% penicillin–streptomycin (Pen–Strep; Gibco, USA). The cell line was obtained from the American Type Culture Collection (ATCC, Cat #CRL-1587).

#### 2.11.2 Cell viability assay by WST-1

The WST-1 assay (Sigma-Aldrich, USA) was performed according to the manufacturer’s instructions (Millipore sigma Protocol Guide: WST-1 Assay for Cell Proliferation and Viability). Briefly, cells were seeded at a density of $5\times 10^{4}$ cells per well in a 96-well microplate and exposed to different concentrations of crude guava leaf extract (3–10 mg/ml). Gentamicin (16 µg/ml) served as the positive control, while cells cultured in medium alone were used as the growth control. The microplate was incubated for 24 h at 37 °C and 5% CO_2_. After incubation, the medium was replaced with 100 µl of fresh culture medium (Takara et al., 2005; Christina et al., 2022). Subsequently, 10 µl of WST-1 reagent was added to each well, and the plate was incubated for four more hours. The plate was then shaken for 1 min, and absorbance was measured at 440 nm using an ELISA reader (Varioskan Lux, Thermo Fisher Scientific, USA). Results are expressed as percentage viability.


$$\%\;Cell\;viability=\frac{Absorbance\;of\;treatment-absorbance\;of\;medium}{Absorbance\;of\;control\;cell-absorbance\;of\;medium\;}\;x\;100$$

(Millipore sigma Protocol Guide: WST-1 Assay for Cell Proliferation and Viability).

#### 2.11.3 Cytotoxicity assay by LDH

The CyQUANT™ LDH Cytotoxicity Assay (Invitrogen, Thermo Fisher Scientific, USA) was performed according to the manufacturer’s instructions (Invitrogen), to corroborate the results obtained in the WST-1 assay. Briefly, cells were seeded at a density of $5\times 10^{4}$ cells per well in a 96-well microplate and exposed to varying concentrations of crude guava leaf extract (3–10 mg/ml), using the same controls as in the WST-1 assay. The microplate was incubated for 24 h at 37 °C and 5% CO_2_. After incubation, 10 µl of lysis buffer was added to the triplicate wells designated as Maximum LDH activity, and the plate was incubated for 45 min. Subsequently, 50 µl of the supernatant from each well was transferred to a new microplate, followed by the addition of 50 µl of reaction mixture. The plate was then incubated for an additional 30 min. Finally, 50 µl of stop solution was added to each well, and absorbance was measured at 490 nm and 680 nm. Results are expressed as percentage LDH release.


$$\%\;LDH\;release=\frac{(Compound-treated\;LDH\;activity)*100}{Maximum\;LDH\;activity}$$

(Kim et al., 2017).

### 2.12 Statistical analysis

Data were analyzed using one-way ANOVA and Tukey *post-hoc* test (alpha=0.05), in Prism (v8.0.1, GraphPad, San Diego, California, USA) and Minitab (v22.1.0.0, State College, Pennsylvania, USA).

## 3 Results

### 3.1 Evaluation of the presence of different phytochemicals

GLE tested positive for all the phytochemical groups analyzed (**Table 1**). The identification of phenolic compounds in this extract has been corroborated in previous studies using UPLC-MS analysis (Gutierrez Montiel et al., 2023).

**Table 1:** Qualitative phytochemical analysis of *Psidium guajava* L. crude leaf extract.

Compound	Phytochemical test and expected result	Result
Saponins	Extract shaking = formation of foam	Positive
Flavonoids	Extract + NaOH 10% = yellow coloration	Positive
Phenolics	Extract + ferric chloride = dark blue coloration.	Positive
Terpenes	Extract + chloroform and sulfuric acid = reddish coloration at the interface.	Positive

### 3.2 Properties of the microencapsulated extract

**Figure 1:**
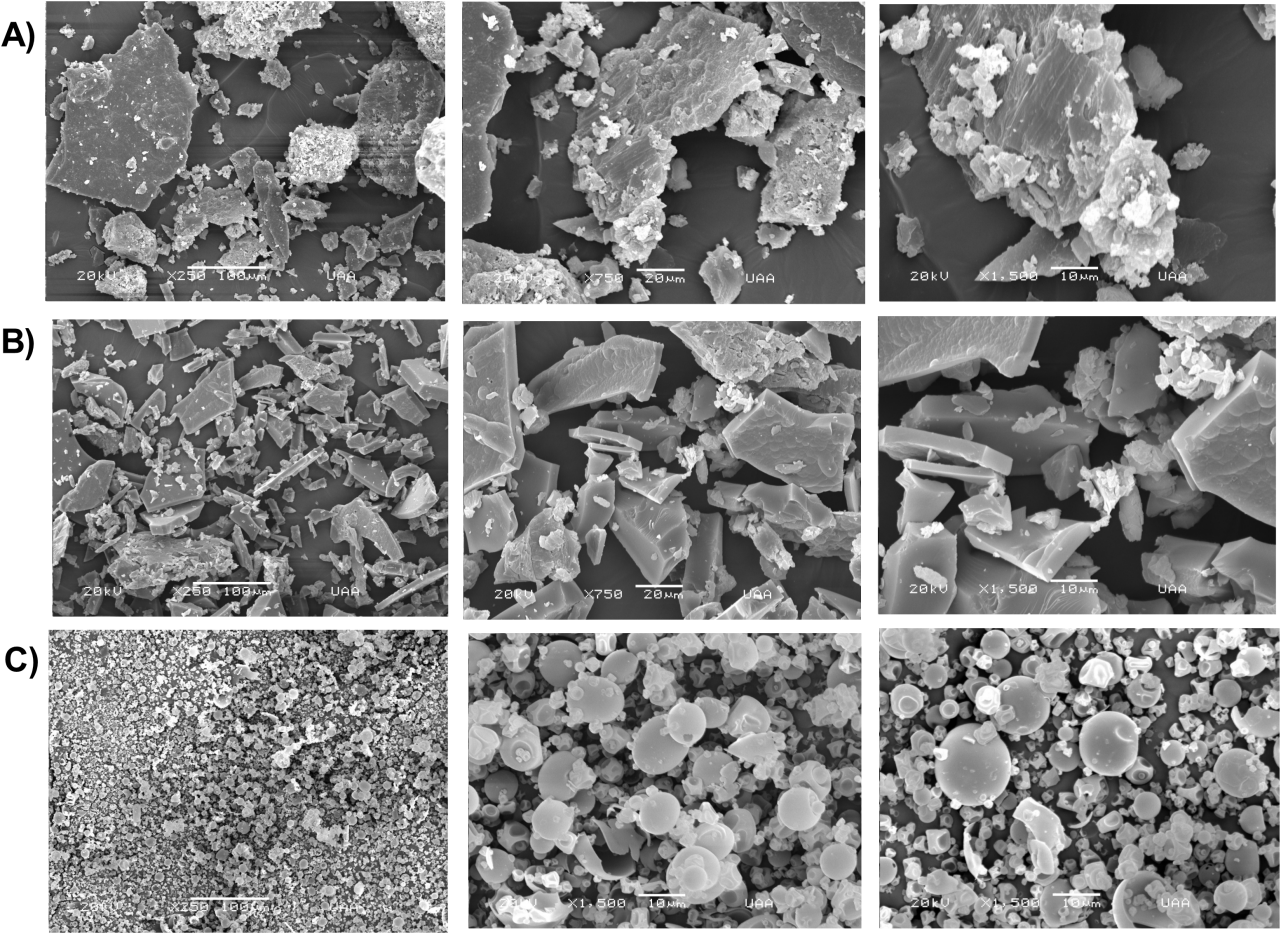
Scanning electron microscopy (SEM) micrographs of: **(A)** crude guava leaf extract, **(B)** purified guava leaf extract, and **(C)** guava leaf extract microencapsulated in β-cyclodextrins.

SEM is commonly used to evaluate the surface morphology of materials and is recognized as a complementary technique for monitoring the formation of inclusion complexes (Escobar-Avello et al., 2021). **Figure 1** shows the micrographs of crude (GLE), purified (GLEP), and microencapsulated (GLEM) guava leaf extracts. As observed, both GLE and GLEP are composed of irregularly shaped particles, whereas GLEM exhibits predominantly spherical particles. This morphological change has also been reported by other authors (Kotronia et al., 2017; Piletti et al., 2019) and is attributed to the loss of crystallinity following the inclusion of the extract into β-cyclodextrins (Escobar-Avello et al., 2021). **Table 2** presents additional characteristics of the microencapsulated extract, including physicochemical properties such as moisture content and solubility, as well as the encapsulation efficiency.

**Table 2:** Characteristics of the microencapsulated guava leaf extract.

Moisture (%)	Solubility (%)	Encapsulation efficiency (%)
3.17 ± 0.21	100 ± 0	79.76 ± 8.54%

The microencapsulated extract showed an encapsulation efficiency of 89%, reflecting effective retention of the active components within the encapsulation matrix. A solubility of 100% indicates good dispersibility in aqueous media, which can facilitate its application. Additionally, a moisture content of 3% suggests acceptable stability, potentially contributing to longer shelf life and reduced degradation risk (Desai and Jin Park, 2005; Tonon et al., 2008; de Jesus Silva et al., 2023).

### 3.2 *E. faecalis* clinical isolate identification

**Figure 2:**
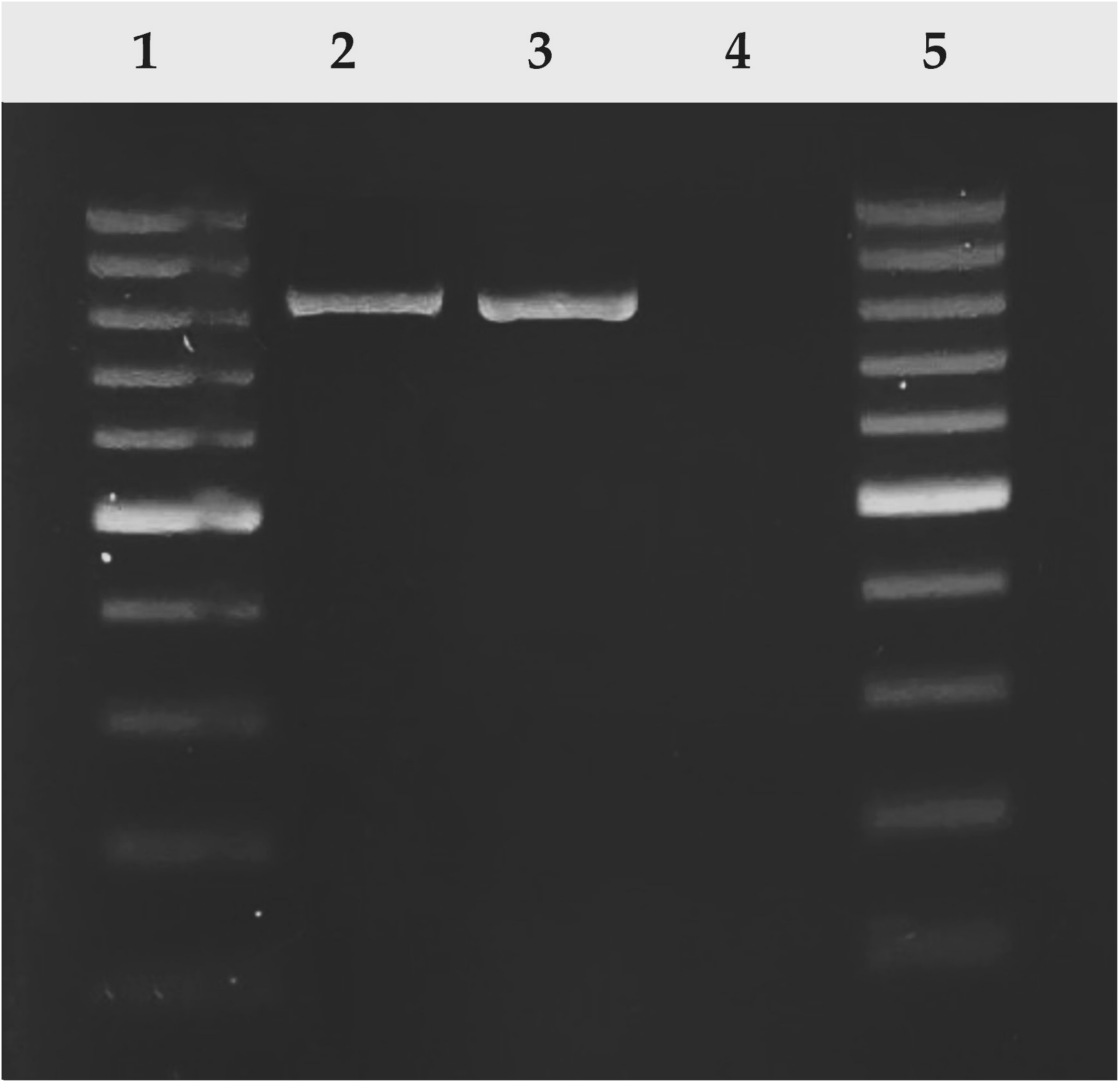
PCR amplification of *E. faecalis* isolate on agarose gel 1.5%. Amplicon 849 bp size was detected in the clinical isolated and positive control (ATCC 29212). Lane 1, 100 pb DNA ladder; lane 2: positive control (*E. faecalis* ATCC 29212); lane 3: *E. faecalis* clinical isolated; lane 4: negative control (water); lane 5, 100 pb DNA ladder.

The clinical isolate was identified as *E. faecalis* through biochemical tests and PCR detection, which employed specific primers targeting position 2528997 to 2529826 of *E. faecalis* chromosome. This yielded a positive amplicon measuring 849 bp (**Figure 2**). Additionally, the isolate was classified as multidrug-resistant as it exhibited resistance to several antibiotics, including ampicillin, tetracycline, doxycycline, clindamycin, gentamicin, and cefotaxime, which are antibiotics of different categories such as beta-lactams, tetracyclines, lacosamide, aminoglycosides, and cephalosporins.

### 3.3 Determination of the minimum inhibitory and minimum bactericidal concentration

**Figure 3:**
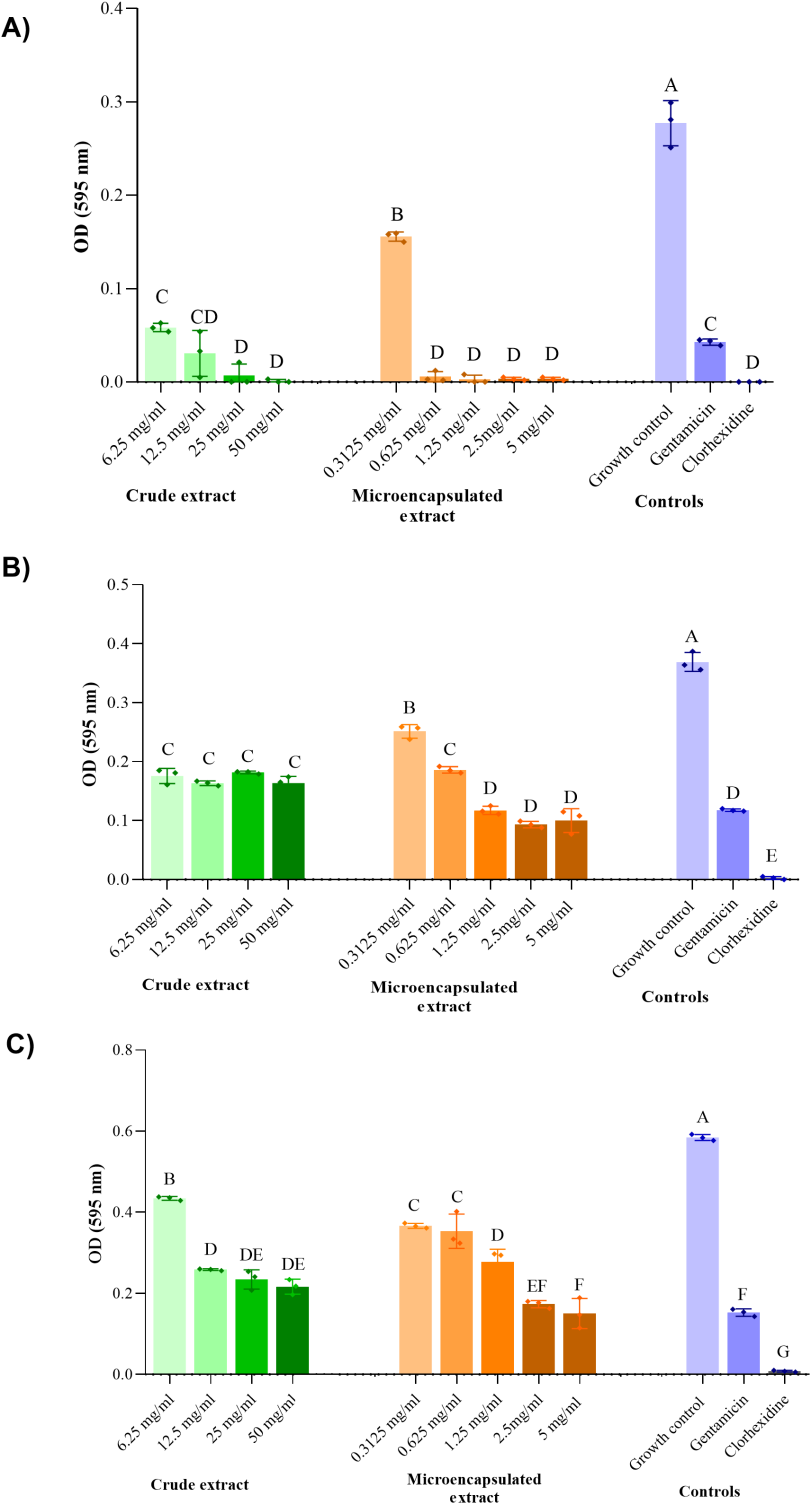
Inhibition of the growth of two strains of *E. faecalis* and one of *S. epidermidis* at different concentrations of crude and microencapsulated guava leaf extract. **(A)***S. epidermidis* ATCC 12228, **(B)** Clinical isolate of *E. faecalis*, **(C)***E. faecalis* ATCC 29212. Conditions that do not share a letter are significantly different (*p* < 0.05).

The results of the MIC and MBC determinations are presented in **Table 3**. The presence of crude guava leaf extract reduced the growth of both *E. faecalis* strains but did not result in complete growth inhibition at the concentrations tested, as shown in **Figures 3B, C**. Therefore, neither a MIC nor an MBC values cloud be determined, since growth occurred on all the agar plates inoculated from the microplate. The microencapsulated extract demonstrated a comparable inhibitory effect, reducing bacterial growth without achieving complete inhibition or exhibiting bactericidal activity. Notably, this effect was observed at ten-times lower concentration than those required for the crude extract.

**Table 3:** MIC and MBC values of guava leaf extract determined for different strains of *E. faecalis* and *S. epidermidis.*

Strain	Gentamicin		Clorhexidin		Crude extract		Microencapsulated	
	MIC	MBC	MIC	MBC	MIC	MBC	MIC	MBC
*Staphylococcus* *epidermidis* ATCC 12228	5 µg/ml	5 µg/ml	0.6%	>0.6%	25 mg/ml	50 mg/ml	0.625 mg/ml	5 mg/ml
*Enterococcus faecalis* ATCC 29212	>5 µg/ml	>5 µg/ml	0.6%	>0.6%	>50 mg/ml	>50 mg/ml	>5 mg/ml	>5 mg/ml
*Enterococcus faecalis* (clinical isolate)	>5 µg/ml	>5 µg/ml	0.6%	>0.6%	>50 mg/ml	>50 mg/ml	>5 mg/ml	>5 mg/ml

MBC= Minimum Bactericidal Concentration.

MIC= Minimum Inhibitory Concentration.

On the other hand, *S. epidermidis* ATCC 12228 was sensitive to the guava leaf extract, presenting complete growth inhibition (**Figure 3A**), with an MIC value of 25 mg/ml and an MBC of 50 mg/ml for the crude extract. Microencapsulation with β-cyclodextrins allowed a reduction in the effective dose, with the minimum inhibitory concentration (MIC) and minimum bactericidal concentration (MBC) determined at 0.625 mg/ml and 5 mg/ml, respectively.

### 3.4 Effect of guava leaf extract on bacterial adhesion

**Figure 4:**
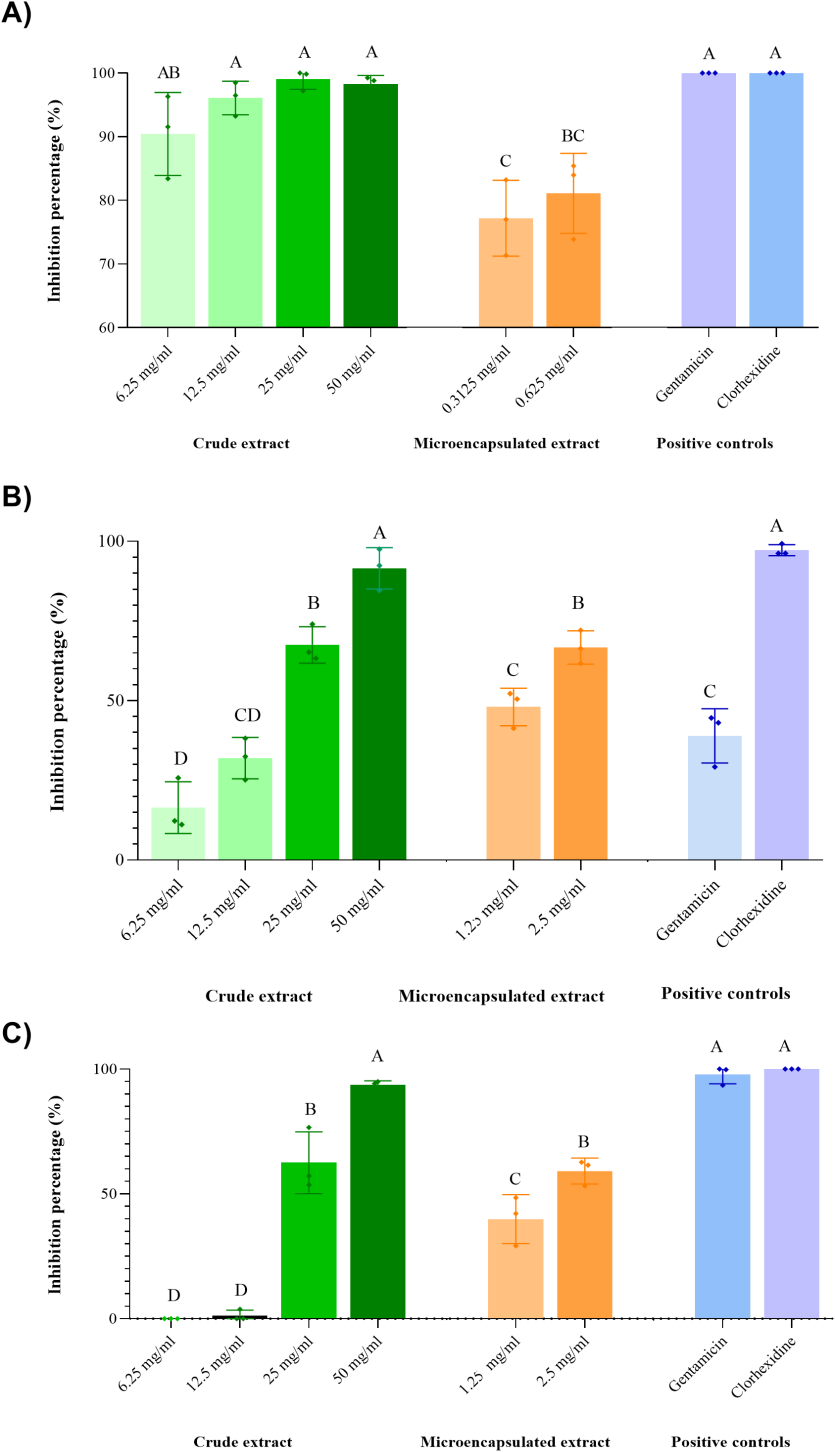
Percentage inhibition of bacterial adhesion at different concentrations of crude and microencapsulated guava leaf extract. **(A)***S. epidermidis* ATCC 12228, **(B)** Clinical isolate of *E. faecalis*, **(C)***E. faecalis* ATCC 29212. Groups that do not share a letter are significantly different (*p* < 0.05).

The results of the anti-adhesion activity of guava leaf extract at different concentration are show in **Figure 4**. In this assay, each bacterium responded differently to the crude guava leaf extract. *S. epidermidis* exhibited almost complete inhibition of adhesion in all the concentrations used. However, it should be noted that at the last two concentrations (25 and 50 mg/ml), the minimum inhibitory and bactericidal concentrations were reached, so the observed effect can no longer be attribute solely to anti-adhesion activity, but rather to antimicrobial activity, as bacterial growth was also inhibited. At the lowest concentration tested (6.25 mg/ml), nearly 100% inhibition of adhesion was observed. This suggests that the extract not only inhibited the growth but also significantly affect the adhesion capacity of *S. epidermidis*. In the case of *E. faecalis* ATCC 29212, greater biofilm formation was observed at 6.25 and 12.5 mg/ml concentrations compared to the negative control. However, at concentration of 25 mg/ml and above, there was more inhibition than 50% of adhesion, with nearly 100% inhibition at 50 mg/ml. Finally, the vaginal clinical isolate of *E. faecalis* had a gradual, concentration-dependent inhibition of adhesion. Although the growth of both *E. faecalis* strains was not completely inhibited by the guava leaf extract, their adhesion and consequently, their biofilm-forming capacity was strongly affected, with almost complete inhibition (nearly 100%) at a concentration of 50 mg/ml.

Furthermore, the microencapsulated extract also reduced bacterial adhesion. In the case of *S. epidermidis* (**Figure 4A**), inhibition rates around 81% and 77% were observed at 0.6 mg/ml and 0.3 mg/ml, respectively. Although these values were slightly lower than those achieved with GLE (90–99% inhibition), it is noteworthy that the GLEM was effective at concentrations ten times lower, underscoring its relevance as an anti-adhesive agent. *E. faecalis* ATCC 29212 exhibited a distinct response depending on the form of the extract (**Figure 4C**). While treatment with GLE at low concentrations led to increased biofilm formation compared to the control, GLEM did not induce this response at the concentrations tested. Instead, it resulted in partial inhibition of adhesion at concentrations of 1.25 and 2.5 mg/ml, with reduction rates of approximately 39% and 59%, respectively. The clinical isolate of *E. faecalis* (**Figure 4B**) was slightly more responsive to GLEM than the ATCC strain, showing inhibition rates of about 48% and 66% at the same concentrations.

### 3.5 Effect of guava leaf extract on biofilm formation by CLSM

**Figure 5:**
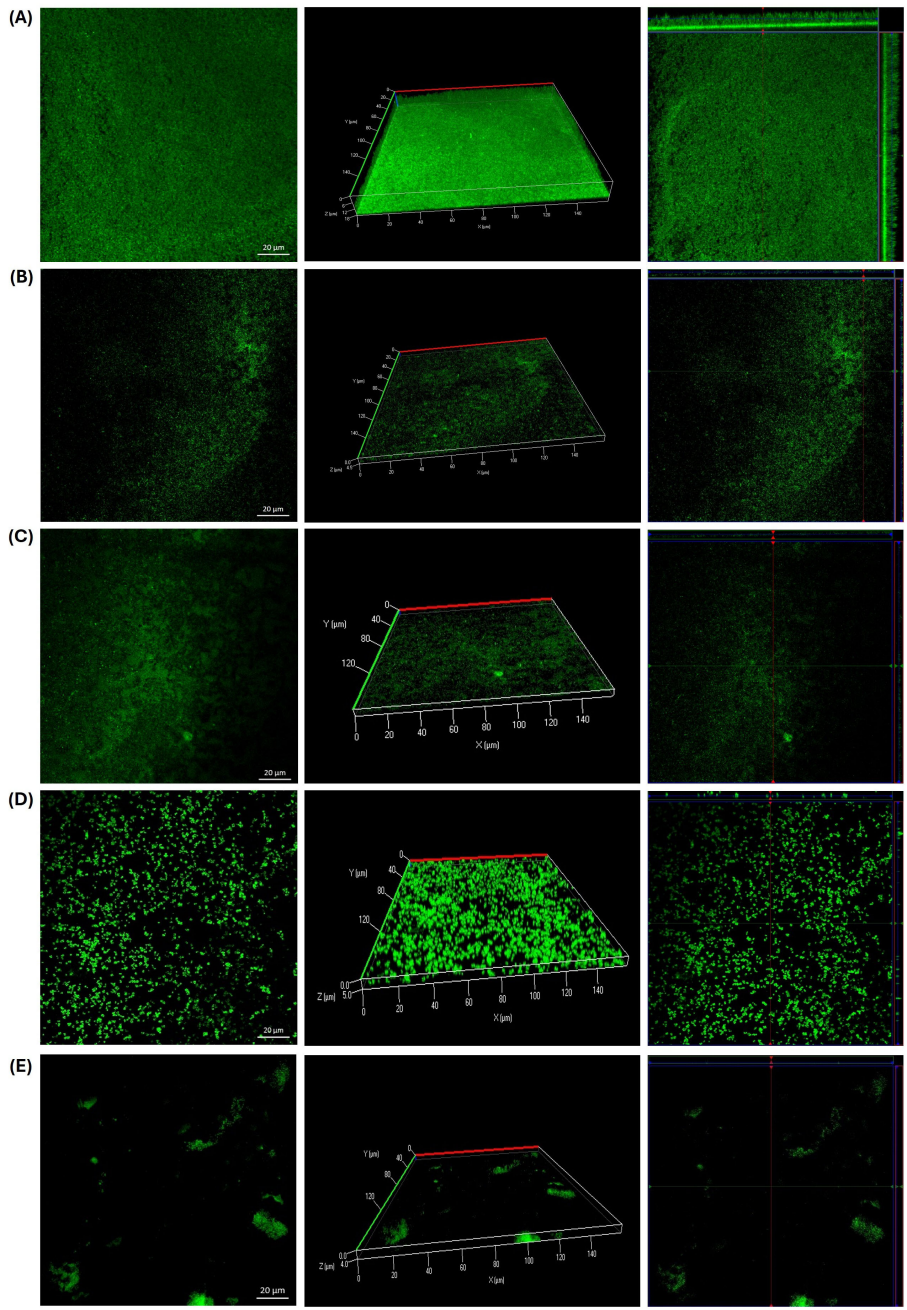
Biofilm formation by *Staphylococcus epidermidis* ATCC 12228 on slides after 24 hours of incubation at 37 °C. **(A)** Growth control. **(B)** Presence of bacterial aggregates following exposure to GLE at 6.25 mg/ml. **(C)** Significant reduction in biofilm formation observed with GLE treatment at 6.25 mg/ml. **(D)** Inhibition of biofilm formation in the presence of GLEM at 0.3125 mg/ml. **(E)** Positive control treated with chlorhexidine at 0.6%.

As shown in **Figure 5**, the presence of guava leaf affected the formation of biofilms by *S. epidermidis*. Both the crude extract (6.25 mg/ml) and the microencapsulated form (0.3125 mg/ml) exhibited similar effects: multiple bacterial aggregates were observed in treated samples (**Figures 5B-D**) along with areas where biofilm formation was notably reduced compared to the untreated control (**Figure 5A**). This resulted in a decrease in biofilm thickness from 18 µm in the growth control to 4.5 µm with GLE and 5 µm with GLEM. These values are comparable to that observed with the positive control, chlorhexidine (0.6%), which showed a thickness of 4 µm (**Figure 5E**).

**Figure 6:**
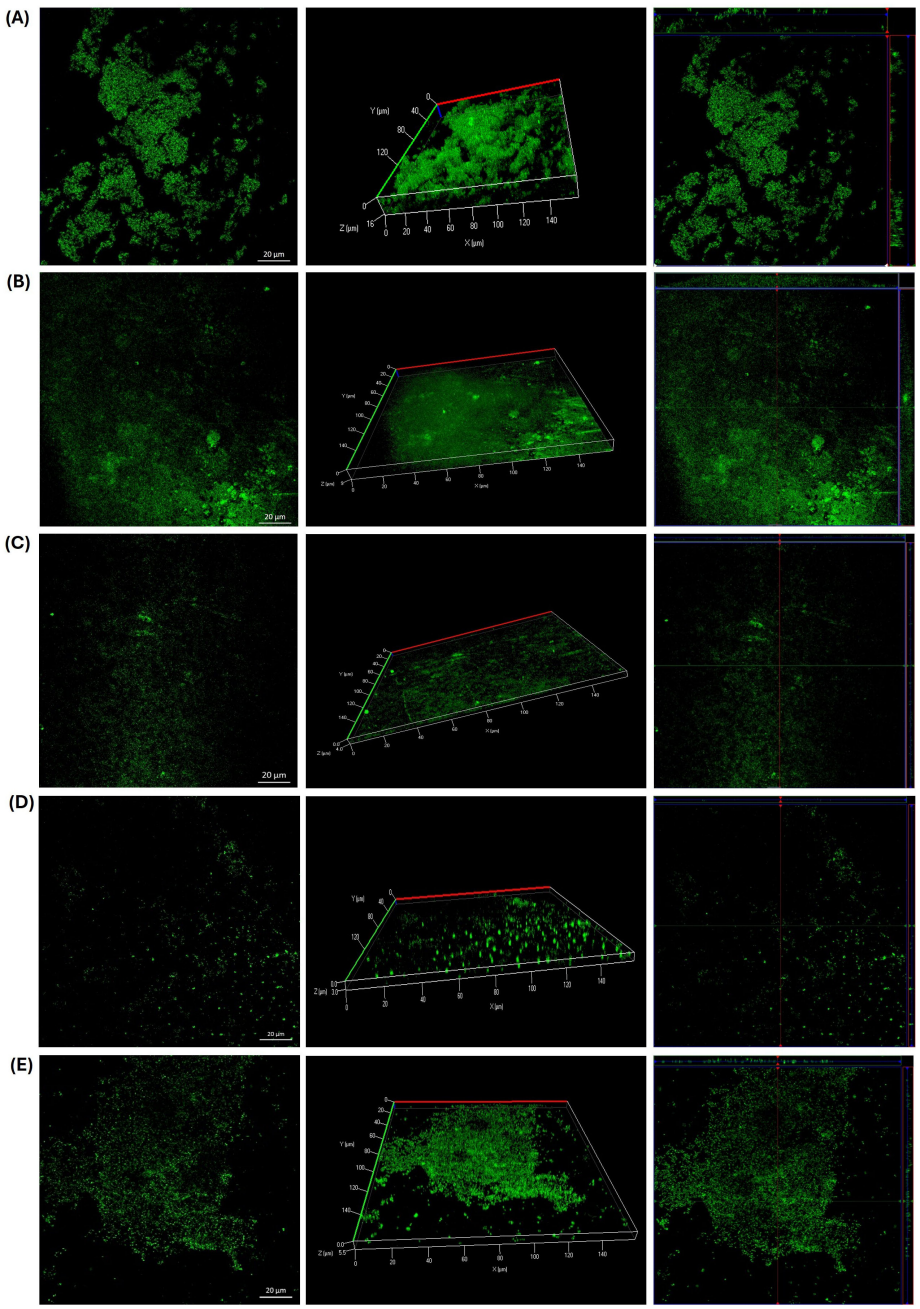
Biofilm formation of a clinical isolate of *E. faecalis* on slides after 24 hours of incubation at 37 °C. **(A)** Growth control. In the slide that was subjected to the presence of GLE (25 mg/ml), bacterial aggregates **(B)** and areas of little bacterial presence with slight biofilm formation **(C)** were observed compared to the growth control. **(D)** Inhibition of biofilm formation in the presence of GLEM at 2.5 mg/ml. **(E)** Positive control treated with chlorhexidine at 0.6%.

**Figure 7:**
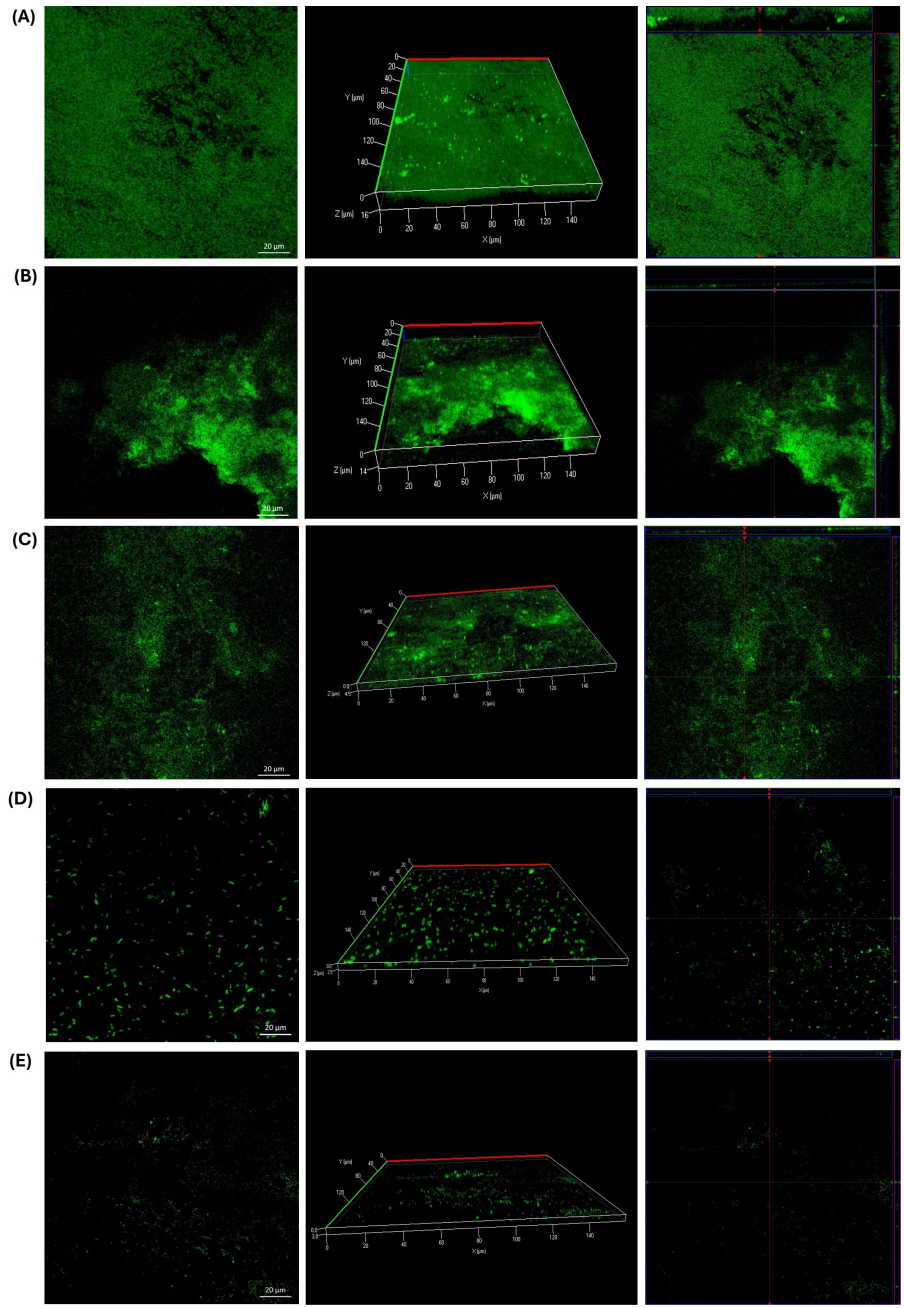
Biofilm formation of *E. faecalis* ATCC 29212 on slides after 24 hours of incubation at 37 °C. **(A)** Growth control. In the slide that was exposed to the presence of GLE (25 mg/ml), bacterial aggregates **(B)** and areas where the adhesion and formation of biofilms decreased considerably **(C)** were observed. **(D)** Inhibition of biofilm formation in the presence of GLEM at 2.5 mg/ml. **(E)** Positive control treated with chlorhexidine at 0.6%.

Comparable effects were observed in both *E. faecalis* strains. Exposure to GLE (25 mg/mL) led to the formation of bacterial aggregates (**Figures 6B**, **7B**); however, these structures displayed reduced thickness compared to the growth controls (**Figures 6A**, **7A**). Additionally, several areas showed reduced biofilm formation, with biofilm thickness decreasing from 16 µm to 4 µm in the clinical isolate, and from 16 µm to 4.5 in the ATCC strain (**Figures 6C**, **7C**). In contrast, treatment with GLEM (2.5 mg/mL) resulted in the absence of large bacterial aggregates, along with a further decrease in biofilm thickness to 3 µm and 2.5 µm in the clinical isolate and ATCC strain, respectively (**Figures 6D**, **7D**). These values were comparable, or, in the case of the clinical isolate superior to those obtained with the positive control, chlorhexidine (0.6%), which produced biofilm thicknesses of 5.5 µm (**Figures 6E**).

**Figure 8:**
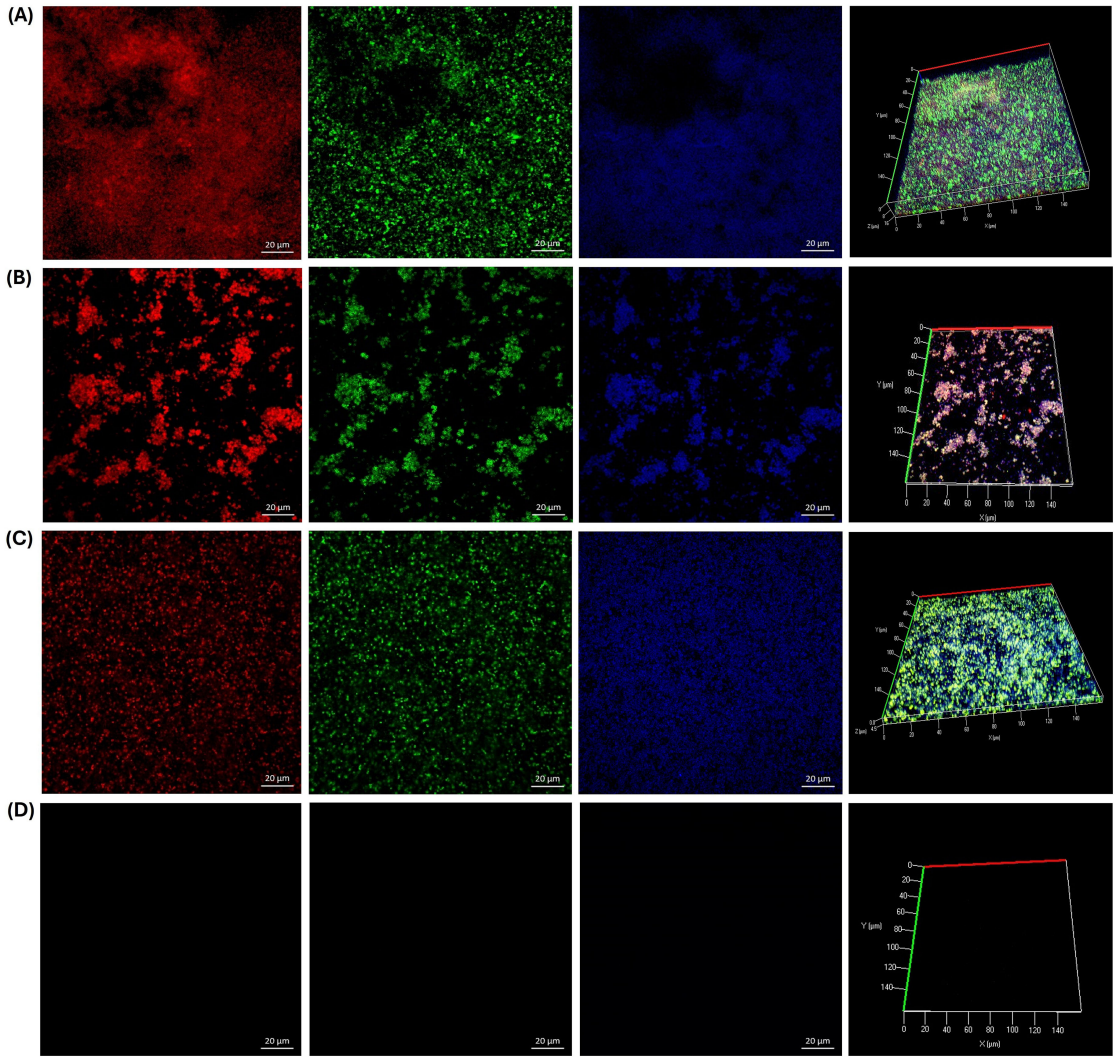
CLSM microscopy of the effect of the guava leaf extract in the crude (GLE) and microencapsulate (GLEM) form on the extracellular polymeric substance (EPS) of the biofilm of *S. epidermidis* ATCC 12228. Proteins were stained with SYPRO Ruby (red), N-acetyl-D-glucosamine and N-acetyl-neuraminic acid residues with wheat germ agglutinin (WGA) conjugated to Oregon Green (green), and extracellular DNA (eDNA) with DAPI (blue). Biofilm development is shown under four conditions: untreated control **(A)**; exposure to crude guava leaf extract at 6.25 mg/mL **(B)**; exposure to microencapsulated extract at 0.3125 mg/mL **(C)**; and treatment with 0.6% chlorhexidine **(D)**.

Additionally, we investigated the effects of GLE and GLEM on the extracellular polymeric substance (EPS) composition of biofilms formed by *S. epidermidis* ATCC 12228, *E. faecalis* ATCC 29212, and a clinical *E. faecalis* isolate. For *S. epidermidis*, the untreated control biofilm (**Figure 8A**) exhibited a dense and compact structure, with abundant protein and polysaccharide signals forming a homogeneous matrix, along with moderate eDNA detection. Exposure to GLE at 6.25 mg/mL (**Figure 8B**) disrupted this architecture, reducing protein and polysaccharide staining and yielding a more heterogeneous distribution, while eDNA became more evident, suggesting cell damage and release of genetic material. Treatment with the microencapsulated extract at 0.3125 mg/mL (**Figure 8C**) also diminished EPS signals, producing a looser and more porous matrix with visible voids compared to the control. Although less pronounced than the effect of crude extract at higher concentration, the reduction in protein and polysaccharide staining demonstrates that the encapsulated form, even at lower dose, retains antibiofilm activity. As expected, chlorhexidine at 0.6% (**Figure 8D**) caused near-complete elimination of biofilm signals, validating its role as a positive control for biofilm eradication.

**Figure 9:**
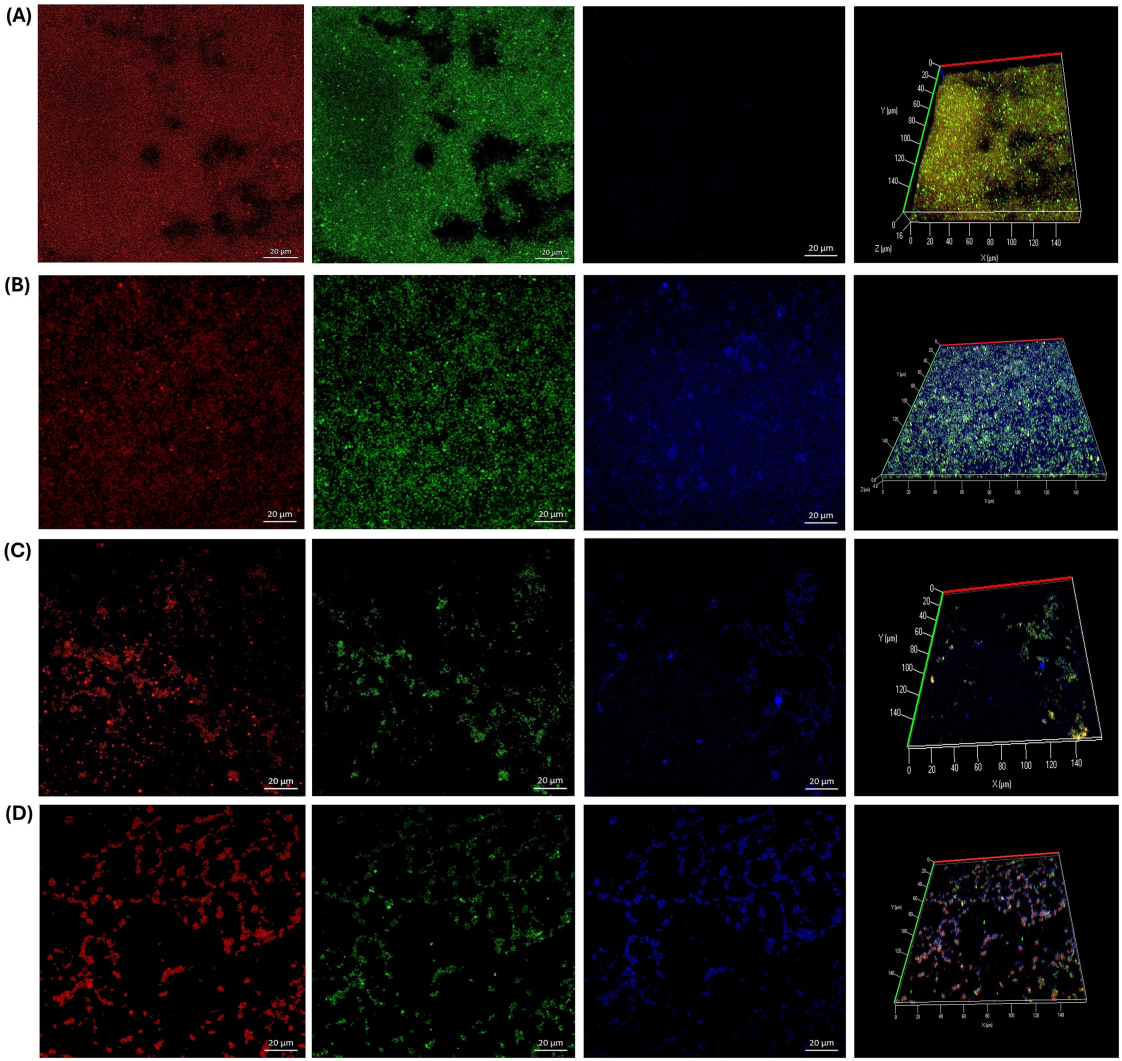
CLSM microscopy of the effect of the guajava leaf extract in the crude (GLE) and microencapsulate (GLEM) form on the extracellular polymeric substance (EPS) of the biofilm of *E. faecalis* clinical isolate extracellular polymeric substance (EPS) of the biofilm. SYPRO Ruby was used to stain proteins (red), wheat-germ agglutinin (WGA)-Oregon green was used to stain N-acetyl-D-glucosamine and N-acetyl-neuraminic acid residues (green) and eDNA was stained with DAPI (blue). Biofilm development is shown under four conditions: untreated control **(A)**; exposure to crude guava leaf extract at 25 mg/mL **(B)**; exposure to microencapsulated extract at 2.5 mg/mL **(C)**; and treatment with 0.6% chlorhexidine **(D)**.

**Figure 10:**
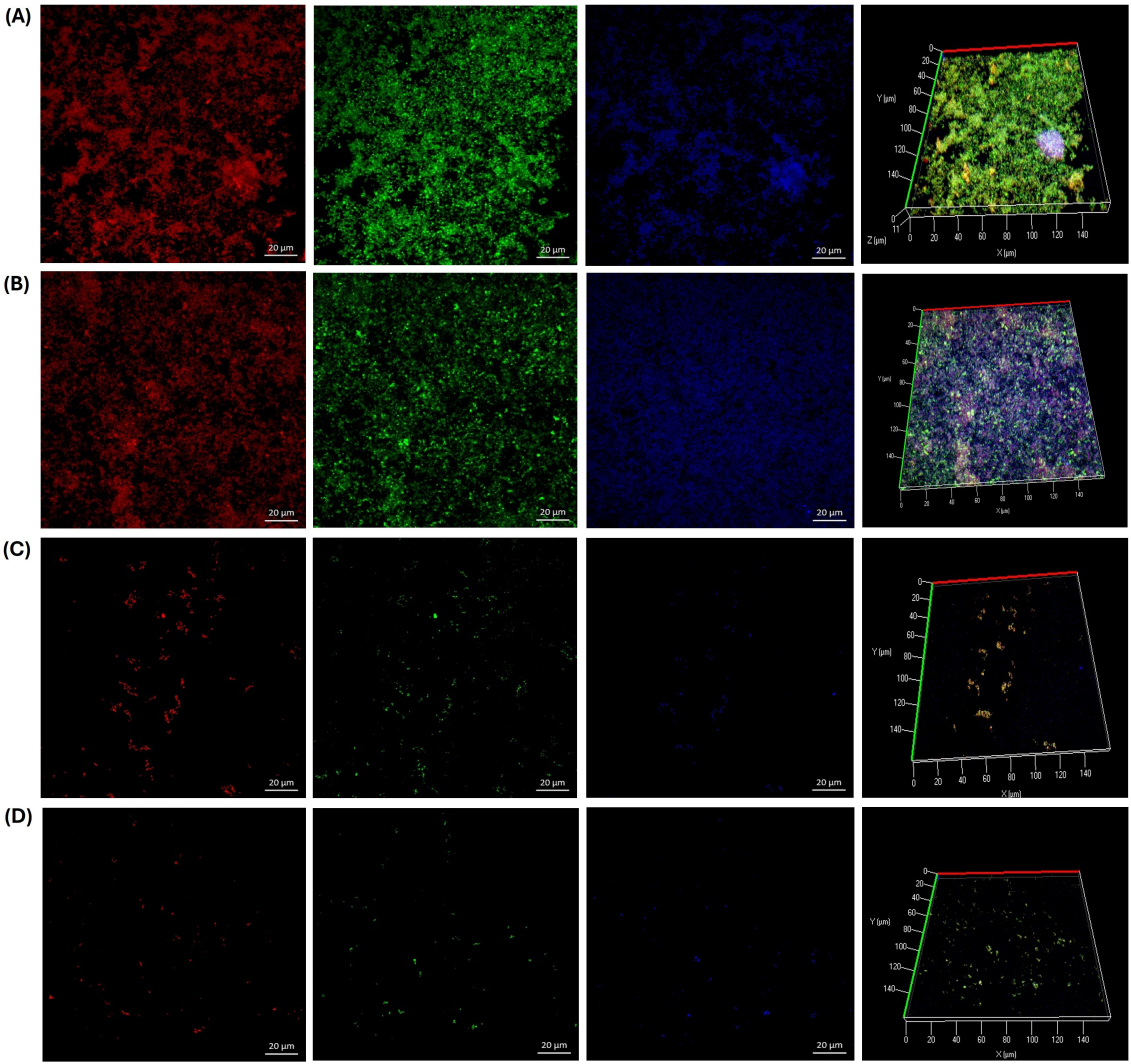
CLSM microscopy of the effect of the guava leaf extract in the crude (GLE) and microencapsulate (GLEM) form on the extracellular polymeric substance (EPS) of the biofilm of *E. faecalis* ATCC 29212. SYPRO Ruby was used to stain proteins (red), wheat-germ agglutinin (WGA)-Oregon green was used to stain N-acetyl-D-glucosamine and N-acetyl-neuraminic acid residues (green) and eDNA was stained with DAPI (blue). Biofilm development is shown under four conditions: untreated control **(A)**; exposure to crude guava leaf extract at 25 mg/mL **(B)**; exposure to microencapsulated extract at 2.5 mg/mL **(C)**; and treatment with 0.6% chlorhexidine **(D)**.

On the other hand, triple fluorescent staining revealed that the treatments had distinct effects on the biofilm matrix of the clinical *E. faecalis* isolate. In the untreated control (**Figure 9A**), the biofilm appeared dense and compact, with strong signals for proteins and PNAG/NANA polysaccharides, and only minor detection of eDNA. Exposure to GLE at 25 mg/mL (**Figure 9B**) resulted in a marked reduction of proteins and polysaccharides, while eDNA became more prominent, indicating partial matrix disruption. Interestingly, the microencapsulated extract at a lower concentration (2.5 mg/mL) caused an even greater reduction of all EPS components, yielding a sparse and discontinuous biofilm. Chlorhexidine (0.6%) induced severe fragmentation, with minimal residual signals. Similarly, in *E. faecalis* ATCC 29212, the untreated biofilm (**Figure 10A**) exhibited a compact and continuous EPS rich in proteins and polysaccharides. Treatment with GLE at 25 mg/mL reduced these signals while increasing eDNA, reflecting disruption of the biofilm structure. Remarkably, the microencapsulated extract at a tenfold lower concentration (2.5 mg/mL) produced an even greater loss of EPS components and structural integrity. Once again, chlorhexidine (0.6%) nearly eliminated all biofilm signals. Finally, it is noteworthy that in all biofilms treated with GLE or GLEM, triple staining revealed a reduction in biofilm thickness comparable to that observed with FilmTracer staining, supporting the disruptive effect.

### 3.6 Effect of guava leaf extract on *Lecane papuana* and *Paramecium caudatum*

In this project, the acute toxicity of the crude leaf extract of *Psidium guajava* L. was evaluated using *L. papuana* and *P. caudatum* as test organisms, as a preliminary approach to assess its potential toxic effects. The results are presented in **Table 4**, where LC_50_, LOEC and NOEC were estimated.

**Table 4:** Acute toxicity assessment of crude *Psidium guajava* L. leaf extract in two model organisms: *Lecane papuana* and *Paramecium caudatum.*

Test organism	Lecane papuana	Paramecium caudatum
$LC_{50}$	3.38 mg/ml	1.27 mg/ml
LC	2.8183-3.9810 mg/ml	1.1357-1.4333 mg/ml
NOEC	0.5 mg/ml	0.5 mg/ml
LOEC	1 mg/ml	1 mg/ml
$R^{2}$	0.83	0.90
CV	11.65	10.36

LC_50_, the concentration at which 50% mortality is observed compared to the control group; LOEC, the Lowest Observed Effect Concentration; NOEC; No Observed Effect Concentration.

In *Lecane papuana*, the LC_50_ was calculated at 3.38 mg/ml, while the no observed effect concentration (NOEC) and the lowest observed effect concentration (LOEC) were 0.5 mg/ml and 1 mg/ml, respectively. In comparison, *Paramecium caudatum* demonstrated higher sensitivity to the extract, with an LC_50_ of 1.27 mg/ml. Despite this increased sensitivity, the NOEC and LOEC values for *P. caudatum* were identical to those of *L. papuana*, suggesting that although both organisms begin to exhibit adverse effects at similar concentrations, *P. caudatum* is more severely affected as the dose increases.

### 3.7 Cell viability and citotoxity assays

In the WST-1 assay, no significant differences in viability were observed for Vero-76 cells treated with guava leaf extract at 3–6 mg/mL, with values ranging from 84.23 ± 4.82% to 95.75 ± 4.80%. A marked reduction in viability was observed only at the highest concentrations, decreasing to 80.92 ± 5.09% at 8 mg/mL and 51.55 ± 9.43% at 10 mg/mL (**Table 5**). Consistently, in the lactate dehydrogenase (LDH) assay, increased LDH release compared with the growth control occurred only at these two highest concentrations (**Table 5**), aligning with the reduction in metabolic activity detected in the WST-1 assay.

**Table 5:** Cell viability (%) and LDH release (%) in Vero-76 cells treated with crude guava leaf extract.

Crude guava leaf extract concentrations (mg/ml)	Cell viability (%)	LDH release (%)
Growth control (0)	100 ± 0*^A^*	29.31 ± 2.20*^A^*
Gentamicin (16 µg/ml)	89.37 ± 4.11*^ABC^*	26.31 ± 1.97*^A^*
3	90.94 ± 4.68*^ABC^*	25.97 ± 1.95*^A^*
4	86.30 ± 3.71*^BC^*	25.62 ± 1.92*^A^*
5	84.23 ± 4.82*^BC^*	28.63 ± 2.15*^A^*
6	95.75 ± 4.80*^AB^*	26.66 ± 2.00*^A^*
8	80.92 ± 5.09 *^C^*	34.93 ± 2.62*^B^*
10	51.55 ± 9.43*^D^*	41.34 ± 3.10*^B^*

Groups that do not share a letter are significantly different (*p* < 0.05).

## 4 Discussion

After the COVID-19, the current global challenge is microbial resistance, which not only represents a significant threat to public health but also creates an economic burden due to higher healthcare costs, prolonged illnesses, and increased hospitalization rates. This exacerbates the social gap, as not all individuals have the economic capacity to cover these costs, especially in developing countries (Barbieri et al., 2017). Furthermore, the rate of new drugs development is much lower compared to the rate at which microbial resistance develops, and side effects usually accompanied these new agents. Consequently, there is an urgent need for novel and improved antimicrobials (Anand et al., 2019; Dadgostar, 2019). Plants are a rich source of a wide variety of phytochemicals such as alkaloids, polyphenols, terpenes and flavonoids, among others, which can exhibit antimicrobial activity through different mechanisms including increased cell permeability, cell wall alterations, inhibition of protein synthesis, DNA damage, inhibition of *quorum-sensing* and hindrance of biofilm formation. This broad diversity of action mechanisms, along with the structural diversity of these compounds, makes them effective even against resistant strains, complicating bacterial resistance acquisition (Al AlSheikh et al., 2020; Takó et al., 2020; Khameneh et al., 2021). *Psidium guajava* L. is a shrub highly available in guava-producing countries such as Mexico, China, and India, has leaves that are often considered undervalued agro-industrial waste. These leaves are rich in phytochemicals particularly polyphenolic compounds (Gutierrez-Montiel et al., 2023). In this study, the antimicrobial activity of both crude and microencapsulated guava leaf extracts was evaluated against *E. faecalis* and *S. epidermidis*, two pathogens of high clinical importance due to their involvement in a large number of nosocomial infections and the increasing reports of multidrug-resistant strains (Chabi and Momtaz, 2019; Guo et al., 2019; Foster, 2020; Wen et al., 2020).

*S. epidermidis* ATCC 12228 was the most susceptible strain tested, exhibiting a minimum inhibitory concentration (MIC) of 25 mg/ml and a minimum bactericidal concentration (MBC) of 50 mg/ml for the crude extract. In contrast, the microencapsulated form demonstrated significantly enhanced activity, with MIC and MBC values of 0.6 mg/ml and 2.5 mg/ml, respectively. Other studies have reported different MIC and MBC values, such as that of Festus, et al (Festus et al., 2020), who determined an MIC value of 20 ± 0.3 mg/ml and an MBC value of 80 ± 0.2 mg/ml using a methanolic extract of guava leaves against a clinical isolate of *S. epidermidis*. On the other hand, Sanches et al. (2005) used an ethanolic guava leaf extract against *S. epidermidis* ATCC 12228, obtaining an MIC value of 250 µg/ml, and an MBC value of 1000 µg/ml. Dela Luna et al. (2024) evaluated the same strain and reported MIC and MBC values of 2.5 mg/ml and 5.0 mg/ml, respectively, using a crude ethanolic extract of guava leaves. Finally, Huang et al. (2021) evaluated the antimicrobial activity of four compounds isolated from guava leaves against *S. epidermidis* ATCC 12228, determining an MIC higher than 32 µg/ml. In contrast, a reduction in the growth of both *E. faecalis* strains was observed with the crude and microencapsulated guava leaf extracts. As seen with *S. epidermidis*, microencapsulation enhanced the antimicrobial effect, achieving comparable bacterial inhibition at doses approximately ten-times lower than those required with GLE. However, MIC and MBC values could not be determined under the tested conditions. Our findings contrast with other studies that report varying levels of antimicrobial activity against *E. faecalis*. For instance, one study reported an MIC of 624 µg/mL for an aqueous guava leaf extract against *E. faecalis* ATCC 29212 (de Araújo et al., 2014). Another study found an MIC of 1.04 mg/mL for a hydroethanolic leaf extract against *E. faecalis* ATCC 4083 (de Carvalho et al., 2025b), while a methanolic bark extract of *P. guajava* L. exhibited an MIC of 6.25 mg/mL against the same strain (Gurmachhan et al., 2020). Moreover, a hydroethanolic extract of *P. guajava* demonstrated a minimum bactericidal concentration (MBC) of 0.52 mg/mL against *E. faecalis* ATCC 29212 and two clinical isolates (de Carvalho et al., 2025a).

It is important to note that MIC and MBC values can vary depending on several factors, such as the bacteria strain evaluated, the extraction conditions (e.g., selected technique, solvent, temperature, time, etc.), environmental influences like climate and soil conditions, as well as methodological differences (Hardege, 1999; Chirinos et al., 2007; Dantas Silva et al., 2017; Lavola et al., 2017). The discrepancies between the values obtained in this study and those reported by other authors are likely attributable to these variables. Moreover, our findings show that microencapsulation of *Psidium guajava* L. leaf extract in β-cyclodextrins significantly improved its efficacy against *Staphylococcus epidermidis* and *Enterococcus faecalis*. This effect has been previously documented in the literature. For example, Rakmai et al. (2018) reported increased antibacterial activity of guava leaf oil against *Staphylococcus aureus* and *Escherichia coli* following encapsulation in hydroxypropyl-β-cyclodextrin (HPβCD). Similarly, Fernandes et al. (2014) developed a *P. guajava* leaf extract via spray drying, using various technological adjuvants, including 8% β-cyclodextrins, and observed superior antimicrobial performance against *S. aureus* and *Candida glabrata* compared to the unencapsulated extract. Although the microencapsulated extract exhibited superior antimicrobial activity, the crude extract also demonstrated relevant and comparable efficacy relative to other plant extracts. For instance, aloe vera and mushroom extracts showed MIC values of 60 mg/ml and 40 mg/ml, respectively, against *E. faecalis* (Kurian et al., 2016). Additionally, extracts of *Leucas aspera* and *Dahlia pinnata* exhibited MIC values of 200 mg/ml against the same bacteria (Saravanan et al., 2021).

The ability of bacteria to adhere to biotic or abiotic surfaces allows them to colonize common areas that we are frequently encounter, such as railings, catheters and handles. This adhesion also allows bacteria to attach to body tissue, facilitating the formation of biofilms and leading to infections (Saxena et al., 2019; Luo et al., 2021). Bacterial adhesion has been recognized as the first stage in biofilm development and is a key step in the process of pathogenesis. Therefore, targeting adhesion becomes an important strategy for eliminating pathogens before a biofilm becomes organized and well-structured (del Carmen Molina Bertrán et al., 2022). We evaluated the anti-adhesion effect of both crude and microencapsulated *Psidium guajava* L. leaf extract using the crystal violet staining method.

In the case of *E. faecalis* ATCC 29212 (**Figure 4C**) a phenomenon not observed with other two bacteria occurred: greater biofilm formation was observed at the two lowest concentrations (6.25 and 12.5 mg/ml) of GLE compared to the control. This may be due to the fact that some microorganisms, under stress conditions, such as the presence of phytochemicals, favor biofilm formation as a survival mechanism (Muhammad et al., 2020; Srinivasan et al., 2021). A similar phenomenon was reported by Negreiros et al (Negreiros et al., 2016), who also observed that sub-inhibitory concentrations of *Baccharis psiadioides* essential oil favored biofilm formation in a clinical isolate of *E. faecalis*, and by Suhartono, et al. (Kotronia et al., 2017), who reported that both low and high concentrations of neem leaf extract induced biofilm formation of a strain of *E. faecalis*. However, this effect was not observed with sub-inhibitory concentrations of GLEM, which demonstrated partial inhibition of adhesion at lower concentrations than GLE, achieving approximately 39% and 59% inhibition at 1.25 mg/ml and 2.5 mg/ml, respectively. As suggested by Christaki et al (Christaki et al., 2023), this may be attributed to the ability of β-cyclodextrin to enhance the aqueous solubility of the extract’s active compounds, thereby facilitating their diffusion through the medium and increasing their accessibility to bacterial cells. Consequently, this improved bioavailability enables effective antibacterial activity at lower extract concentrations. This is also consistent with the observations made in the determination of MIC and MBC. Furthermore, other authors have suggested that β-cyclodextrin may enhance penetration through the bacterial outer membrane, potentially disrupting adhesion mechanisms. Additionally, cyclodextrins might interfere with quorum sensing by interacting with autoinducers (Saha and Rafe, 2023; Alabrahim et al., 2025). These proposed mechanisms could collectively contribute to preventing bacterial adaptation at sub-inhibitory concentrations. However, more studies are required to clarify the exact mechanism by which microencapsulation with cyclodextrins enhances antimicrobial and antibiofilm activities.

Although multiple studies reported the antimicrobial activity of *Psidium guajava* L. extracts against *E. faecalis* (Dubey, 2016; Mukunda, 2019; Baldoni et al., 2023) and *S. epidermidis* (Fagbohun et al., 2013; Wang et al., 2017), to our knowledge, there are just a few reports on its anti-adhesion or antibiofilm activity against these microorganisms. Among them, de Carvalho et al. (2025a) reported that a hydroethanolic guava leaf extract demonstrated effectiveness in reducing the viability of biofilms formed by clinical strains of *E. faecalis*, with inhibition rates ranging approximately from 41% to 55% at concentrations of 1.04 mg/ml and 0.52 mg/ml. Moreover, relevant inhibition percentages with both, GLE and GLEM, were obtained when compared with those obtained for other plant extracts. For example, Negreiros et al. (2016) reported biofilm formation inhibition percentages of 17.1% for *E. faecalis* ATCC 29212 and 75.8% for *S. epidermidis* ATCC 35984, using the MIC concentration of *Baccharis psiadioides* essential oil. Suhartono et al. (2023) reported an inhibition of 36.85% for a strain of *E. faecalis* using a 12.5% concentration of neem leaf extract. Linda, et al. (Rochyani, 2021) reported an inhibition percentage of 55.78 ± 3.68% using an 80% concentration of *Moringa oleifera* extract. Martínez Chamás et al. (2023) reported an inhibition of 66.56 ± 0.43 for a *S. epidermidis* isolate treated with 1/8 × MIC of *Fabiana densa* extract, while Di Lodovico et al. (2020) achieved an inhibition of 86.96% for *S. epidermidis* ATCC 12228 at the MIC of hop extract.

Furthermore, the results showed that the presence of guava leaf extract affected the biofilm formation by reducing their thickness in all strains tested on CLSM analysis. It should be noted that, in all three microorganisms studied, the same phenomenon was observed: the formation of bacterial cells aggregates in the presence of GLE, compared to the growth control (**Figures 5B**, **6B**, **7B**), a finding also observed with the naked eye. This phenomenon has already been reported by other authors, including Wolinsky et al (Wolinsky et al., 1996), who observed that incubation of oral *streptococci* with neem stick extract led to microscopically visible bacterial aggregation, reducing the ability of some of them to colonize the surface of teeth. Lee, et al (Lee et al., 2014), reported that the presence of soy extract increased bacterial agglutination, decreasing the adhesion of bacteria, including *Streptococcus mutans*, to orthodontic wire. Similarly, Wang et al (Wang et al., 2021), observed that oolong and pu-erh tea extracts influenced *streptococcal* autoaggregation, resulting in a reduction of adhesion.

Although autoaggregation is an important and advantageous physical interaction for biofilm development, excessively large aggregates and cell clusters are particularly susceptible to physical or chemical detachment, which can lead to their subsequent removal (Zakaria Gomaa, 2013; Wang et al., 2021). This may explain and corroborate what was observed in the microplate assay, where aggregates that may have formed were possibly easily removed during the washes along with the planktonic cells. In the slide test case, however, the aggregates were not eliminated, as the first step was heat fixation. It has also been reported that bacteria autoaggregation is a protective response to external stress, which could represent a risk, as it could protect the bacteria from the host immune system or from antibiotics (Trunk et al., 2018). Therefore, further studies are needed to evaluate the viability and biofilm formation capacity of these aggregates and the potential risks they could represent. While the potential flocculating effect of guava leaf extract may present challenges if aggregates accumulate on the surface, its presence can be beneficial for removing microorganisms due to their reduced adhesion (Nwoko and Okeke, 2021).

The previously described autoaggregation effect was less pronounced when the strains were exposed to GLEM. In this case, bacterial aggregates were fewer and smaller compared to those observed following exposure to GLE. Nevertheless, a marked reduction in biofilm thickness was also observed. This outcome may be attributed to alternative mechanisms by which phytochemicals exert anti-adhesion activity, including inhibition of quorum sensing, blockage of adhesins and other surface-associated proteins, reduced bacterial motility, decreased production of exopolysaccharides, and consequently, the formation of a weaker extracellular matrix (Mishra et al., 2020; Menkem, 2022; Gonçalves et al., 2023; Silva et al., 2023). In a previous study, we reported that phytochemicals in the purify guava leaf extract is composed of vescalagrin, quercetin glucuronide, catechin, casaurinin/causaurictinlsomer, reynoutrin, guajaverin, avicularin, myrciaphenone B, guavinoside C, guavinoside B, leutelin 7-O malonyl-glucoside, kaempferol 3-(6''-malonylglucoside), chryoseriol 7-O- (6′′-malonyl-glucoside), among other compounds that were not identified (Castro-López et al., 2019). These compounds are present in the microencapsulated form as well as are part of the crude extract.

For example, the hydrolysable tannin vescalagrin modulates the assembly of peptidoglycans on bacterial surfaces, disrupting cell walls, causing bacterial cell death, and preventing biofilm formation in *S. epidermidis*, *S. aureus*, and *P. aeruginosa* (Araújo et al., 2021). Ellagitannins like casuarinin and casuarictin inhibit biofilm adhesion by *Candida albicans* through the precipitation of proteins involved in biofilm formation (Glasenapp et al., 2019). Quercetin derivatives, such as reynoutrin, exhibit antibacterial activity and enhance the effectiveness of the antibiotic oxacillin against *S. epidermidis* and methicillin-resistant *S. aureus* (de Jesus Borges Costa et al., 2025). Luteolin 7-O malonyl-glucoside suppresses biofilm formation in *Pseudomonas*, *Salmonella* Typhimurium, and *Serratia marcescens* by disrupting cell membranes and inhibiting bacterial communication (Žemlička et al., 2014). It also damages the membranes of *Escherichia coli*, reducing ATP synthesis and downregulating resistance genes (Ding et al., 2024). Additionally, luteolin can disperse preformed biofilms of *C. albicans* and *E. faecalis*, enhancing antimicrobial treatment by blocking biofilm formation and reducing exopolysaccharide and protein yields (Fu et al., 2021). Chrysoeriol 7-O-(6''-malonyl-glucoside) disrupts the cytoplasmic membrane of multidrug-resistant *Vibrio cholerae* (Tagousop et al., 2018). Guajaverin, decrease the hydrophobicity of *Streptococcus mutants*, which is an initial factor for the oral pathogenic bacteria to adhere to the tooth surface, possibly binding to cell-surface proteins reducing the overall cell hydrophobicity (de Jesus Borges Costa et al., 2025). The kaempferol 3-O-(6′′-malonyl glucoside) compound, inhibited the attachment phase of biofilm formation of *S. aureus* by the reduction of the activity of *S. aureus* sortaseA (SrtA) and the expression of adhesion-related genes, including *clfA* and *clfB*, which encode clumping factor A (ClfA) and ClfB and down-regulated *fnbA* and *fnbB* genes, which encode fibronectin-binding proteins (FnbpA and FnbpB) (Ming et al., 2017; Periferakis et al., 2022).

Flavonoids, including catechins like epigallocatechin-3-gallate (EGCG), proanthocyanidins, quercetin, and myricitrin, have been shown to reduce biofilm formation in several bacteria (Lee and Tan, 2015; Trentin et al., 2015; Motlhatlego et al., 2020; Yang et al., 2020). EGCG can kill bacteria within biofilms and eliminate established biofilms on *E. faecalis* (Lee and Tan, 2015). Catechins can reduce biofilm formation by *Alcaligenes faecalis* and *Pseudomonas gingivalis* by interfering with quorum sensing (Lahiri et al., 2021). Quercetin can decrease *E. faecalis* biofilm formation by 75-90% at sub-inhibitory concentrations. It disrupts protein synthesis and translation pathways by inhibiting specific enzymes and stimulates oxidative stress responses in *E. faecalis* (Qayyum et al., 2019). Additionally, quercetin may exert antibacterial effects through metal chelation and the inhibition of DNA topoisomerase (Liu and Guo, 2015). Although studies on guavinoside C, guavinoside B, and myrciaphenone B are limited, they may also contribute to the extract’s antimicrobial activity. Overall, the extract’s antimicrobial and anti-adhesion effects likely involve shared mechanisms affecting both *S. epidermidis* and *E. faecalis*.

The extracellular polymeric substance (EPS) is essential for maintaining biofilm integrity, protecting it from desiccation, UV radiation, and other environmental stressors. In addition, variations in EPS composition, influenced by environmental conditions, play a critical role in defining the structural and functional characteristics of the biofilm (Minich et al., 2022). EPS are primarily composed of polysaccharides and proteins, both of which play essential roles in biofilm formation and protection. Proteins aid microbial colonization and stress defense, while polysaccharides facilitate adhesion, maintain biofilm structure, and create nutrient gradients that support heterogeneous, resilient microbial communities (Rusanowska et al., 2019). Specifically, Poly-β-1,6-*N*-acetyl-d-glucosamine (PNAG) was originally described as polysaccharide intercellular adhesin (PIA) and appears to be a major constituent of many biofilms, including those formed for *Staphylococcus epidermidis* and *E. faecalis* (Gökçen et al., 2013; Ramos et al., 2019). PNAG contributes significantly to biofilm stability as well as providing resistance to host immune responses and antimicrobial agents (Balducci et al., 2023). Finally, extracellular DNA (eDNA) is a also a crucial component of the EPS. Although it was historically considered less essential, eDNA is now recognized for its role in promoting initial cell adhesion and maintaining the structural integrity of the biofilm matrix (Campoccia et al., 2021).

In our study, a triple fluorescent staining approach was employed to investigate the effect of guava leaf extract on the composition of extracellular polymeric substances (EPS): SYPRO Ruby was used to stain proteins (red), wheat-germ agglutinin (WGA)-Oregon Green 488 stained N-acetyl-D-glucosamine (PNAG) and N-acetyl-neuraminic acid (NANA) residues (green), and extracellular DNA (eDNA) was stained with DAPI (blue). Across all three bacterial strains, a decrease in protein and polysaccharide signals was observed, accompanied by an increase in eDNA detection and a reduction in biofilm thickness, both in the presence of crude and microencapsulated extracts. Notably, for the two *E. faecalis* strains, the microencapsulated extract induced a greater disruption of the biofilm, despite being applied at a tenfold lower concentration (2.5 mg/mL) compared to the crude extract (25 mg/mL).

Similar findings have been reported by other authors. For instance, Fu et al. (2021) observed that lutein, a natural carotenoid, reduced protein and polysaccharide levels within biofilms of *E. faecalis* 20033. Ali et al. (2021) reported a significant reduction in exopolysaccharides of biofilms formed by *E. faecalis* OG1RF in the presence of trans-cinnamaldehyde, an aromatic aldehyde predominantly found in cinnamon essential oils. Conversely, Minich et al. (2022) reported that vanillin and syringic acid exhibited inhibitory effects on PNAG content in the EPS of two clinical isolates of *S. epidermidis* and *S. epidermidis* ATCC 35984, but did not affect protein levels. In contrast, our study observed a reduction in both polysaccharides and proteins.

**Figure 11:**
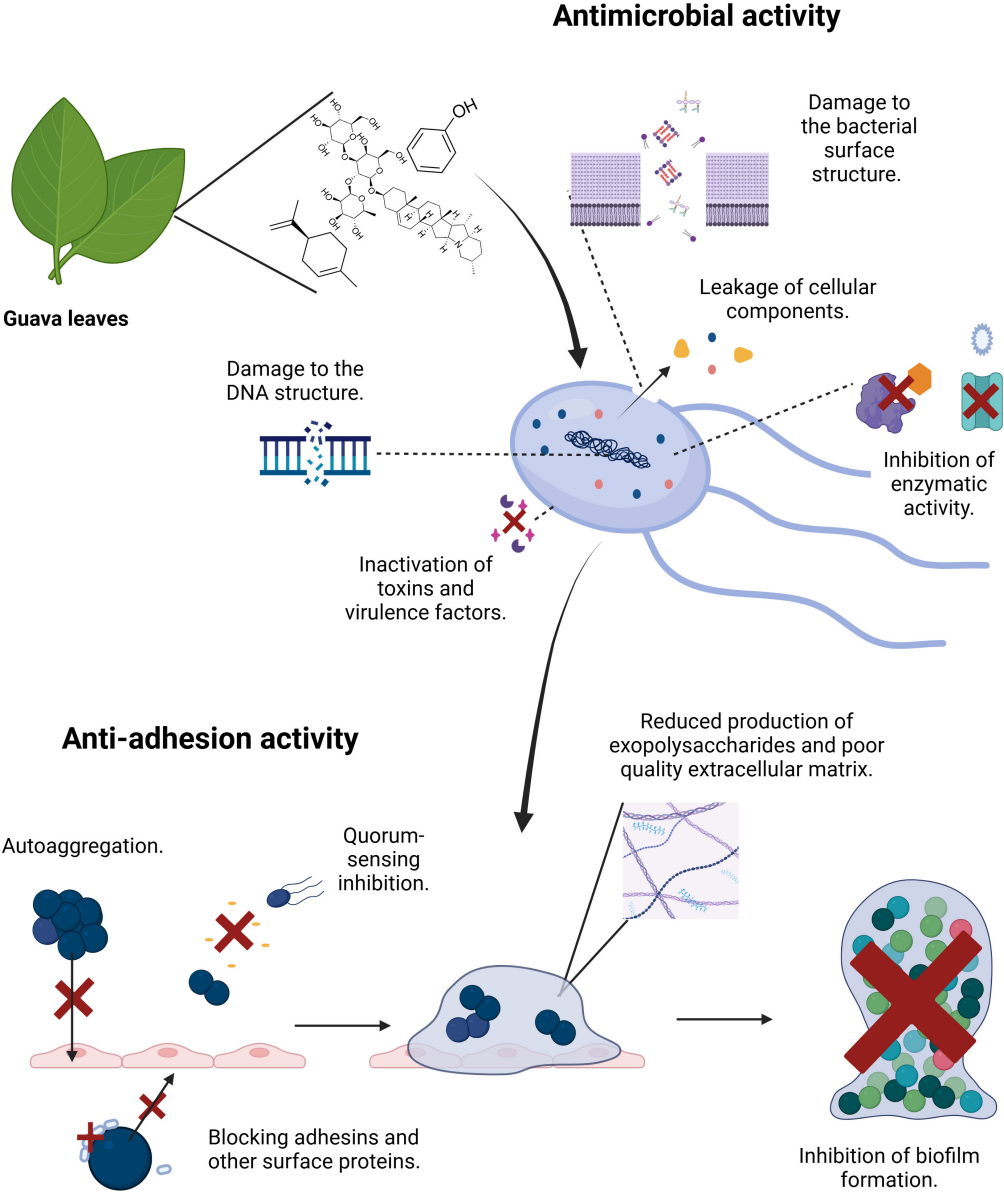
Potential mechanisms of action by which guava leaf extract exhibit antimicrobial and anti-adhesion activity. Adapted and modified from Gonçalves, et al (Gonçalves et al., 2023).

The observed increase in eDNA is noteworthy, as it likely reflects a stress-induced response of the bacterial cells to the guava leaf extract. While eDNA contributes structurally to biofilms, its accumulation in the context of reduced proteins and polysaccharides suggests a destabilized matrix. However, this phenomenon may represent a complex effect, as elevated eDNA could potentially enhance biofilm formation and contribute to antimicrobial resistance (Tahrioui et al., 2019; Sharma and Rajpurohit, 2024). Further studies are therefore required to elucidate the precise implications of eDNA accumulation in treated biofilms and its impact on bacterial survival and resilience. Overall, guava leaf extract, whether crude or microencapsulated, altered the composition of extracellular polymeric, thereby generating a structurally compromised biofilm matrix. **Figure 11** schematizes the potential mechanisms by which the phytochemicals present in guava leaf extract can exert antimicrobial and anti-adhesion activity. Nevertheless, further studies are necessary to elucidate the precise mechanisms by which this natural extract acts against these bacteria.

The acute toxicity of GLE was evaluated using *L. papuana* and *P. caudatum* as test organisms. Rotifers are cosmopolitan, aquatic or semi-aquatic microscopic invertebrates that have been used as models in the detection of chemical substances and environmental samples. To do so, they must be abundant, native and/or representative of the ecosystem and of ecological importance (Rico-Martínez et al., 2016). The *Lecane* genus is one of the largest representatives among freshwater rotifers in Mexican territory, occupying up to 45% of rotifer species, having a presence in the central and southern states of Mexico, and it also has a wide distribution and can be found from the United States to Brazil (Aguilar et al., 2019). On the other hand, protozoa are often used as bioindicators of chemical contamination, especially in aqueous environments, and, among protozoa, *P. caudatum* is one of the most used ciliate models for laboratory research (Venkateswara Rao et al., 2007). Although there are no studies that have used the same model organisms to test the acute toxicity of guava leaf extract, there are studies with other invertebrates, especially with *Artemia salina*, a crustacean widely used in tests to determine the toxicity of natural products (Nguta, 2012). Among them, Bautista et al. (2018) determined an LC_50_ of 0.929 mg/ml for the aqueous extract of guava leaves; Lee et al. (2013) published an LC_50_ value of 1.0009 mg/ml for the essential oil of *P. guajava* L. bark. For our part, an LC_50_ value equal to 1.27 mg/ml was reported for *Paramecium caudatum*, and 3.38 mg/ml for *Lecane papuana*. It should be noted that P*. caudatum* was more sensitive to the presence of guava leaf phytochemicals since its LC_50_ value is lower.

Finally, in this study, we evaluated cell viability using both the WST-1 and LDH assays in African green monkey kidney cells (Vero cell lines), which are widely recommended for *in-vitro* chemical toxicity studies and are considered a standard model for assessing general cytotoxicity in mammalian cells (Fernández Freire et al., 2005; Perryman et al., 2018). We found that crude guava leaf extract at concentrations of 1–6 mg/mL did not affect metabolic activity or LDH release. In contrast, a reduction in metabolic activity and an increase in LDH release were observed at concentrations of 8 mg/mL and above.

It is important to note that both the LC_50_ values obtained in *L. papuana* and *P. caudatum*, as well as the concentrations at which changes were observed in Vero-76 cells, are significantly lower than the effective concentrations of GLE, highlighting the need to explore strategies to mitigate its toxicity. One promising approach is microencapsulation, which has been reported to enhance the long-term stability of phytochemicals, improve their bioavailability, and reduce toxicity (dos Santos Lima et al., 2019; Martins et al., 2021; Nicolaescu et al., 2025). Therefore, evaluating the toxicity of microencapsulated guava leaf extract (GLEM) is proposed as an important objective for future research.

In this study we demonstrate the ability of *Psidium guajava* L. extract to significantly reduce the biofilm biomass of *Staphylococcus epidermidis* ATCC 12228, *Enterococcus faecalis* ATCC 29212, and a clinical vaginal multidrug-resistant isolated of *Enterococcus faecalis.* Indeed, we were able to establish the antimicrobial activity of the extracts with MIC values of 0.625 mg/ml for the microencapsulated extract and of 50 mg/ml for the crude form against *S. epidermidis*. For *E. faecalis*, although it was not possible to determine a MIC at the concentrations tested, both GLE and GLEM induced a statistically significant reduction in bacterial growth and biofilm formation. The purified bioactive compounds in the extract of *Psidium guajava* L. have been previously characterized. These compounds are present in both the crude and microencapsulated extracts, allowing us to hypothesize potential mechanisms for their action. For instance, they may disrupt bacterial cell membranes and prevent initial attachment, thereby inhibiting biofilm formation.

However, this study has some limitations. Firstly, there was a lack of chemical characterization of the bioactive compounds in the crude extract, as well as a quantitative analysis to correlate specific compounds with their observed effects. Future research should address these parameters to gain a better understanding of the physicochemical properties that influence the observed antimicrobial activity. Also, the absence of quantitative analysis of the confocal images represents a methodological limitation. However, the qualitative interpretation of these images allowed for a visual characterization of the treatment’s effects on biofilm architecture, which complements the quantitative data obtained through other assays. Additionally, evaluating the cytotoxicity of the microencapsulated extract and utilizing proteomics to thoroughly elucidate the underlying mechanisms is recommended. Furthermore, it would be beneficial to explore potential synergies with other antimicrobials and assess their effectiveness as disinfectants.

## 5 Conclusions

Guava leaf extract exhibited antimicrobial and anti-adhesion activity against *Staphylococcus epidermidis* ATCC 12228, with a minimum inhibitory concentration (MIC) of 25 mg/ml for the crude extract and 0.625 mg/ml for the microencapsulated form. In contrast, a moderate effect was observed against *Enterococcus faecalis* ATCC 29212 and a clinical vaginal isolate, for which neither MIC nor minimum bactericidal concentration (MBC) could be determined within the tested concentration range. However, both strains showed statistically significant reductions in bacterial growth and adhesion. Furthermore, Both GLE and GLEM reduced protein and polysaccharide content while increasing eDNA within the extracellular polymeric substances suggesting that the extracts may compromise the structural stability of the biofilm matrix. Encapsulation with β-cyclodextrins enhanced the biological activity, allowing for a considerable reduction in the effective dose. The toxicity assessment of the crude extract using *Lecane papuana*, *Paramecium caudatum* and Vero-76 cells revealed that GLE can exert cytotoxicity effects at concentrations lower than those required for antimicrobial activity, pointing the need of optimizing its safety for potential applications. Future studies should evaluate the extract’s antimicrobial and anti-adhesion activity across a broader range of bacterial strains, particularly multidrug-resistant clinical isolates. Additionally, a more in-depth assessment of cytotoxicity is required, including testing in diverse biological models, to better understand its safety profile and therapeutic potential. In addition, exploring potential synergistic effects between the extract and conventional antibiotics, disinfectants, or other plant-derived compounds may contribute to the development of more effective antimicrobial strategies.

## Data Availability

The original contributions presented in the study are included in the article/**Supplementary Material**. Further inquiries can be directed to the corresponding author.
