# Supplementary figures and images for: Antimicrobial and antibiofilm effects of crude and microencapsulated guava leaf extracts against Enterococcus faecalis and Staphylococcus epidermidis
Source: Front Antibiot. 2025 Dec 3;4:1615787. doi: 10.3389/frabi.2025.1615787 (PMC12708567; doi:10.3389/frabi.2025.1615787)

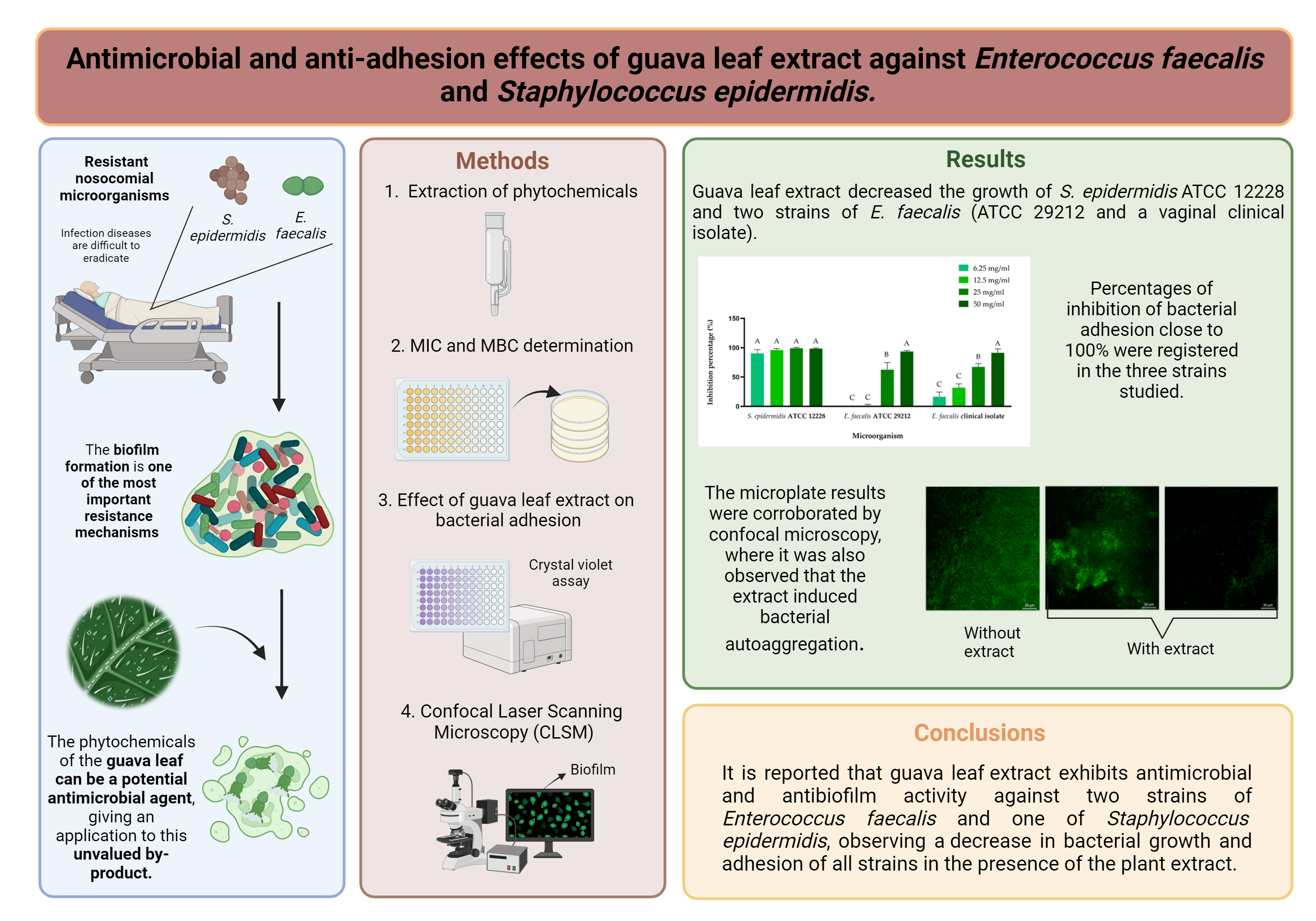

Supplement: Supplementary file 1 [file Image1.png]

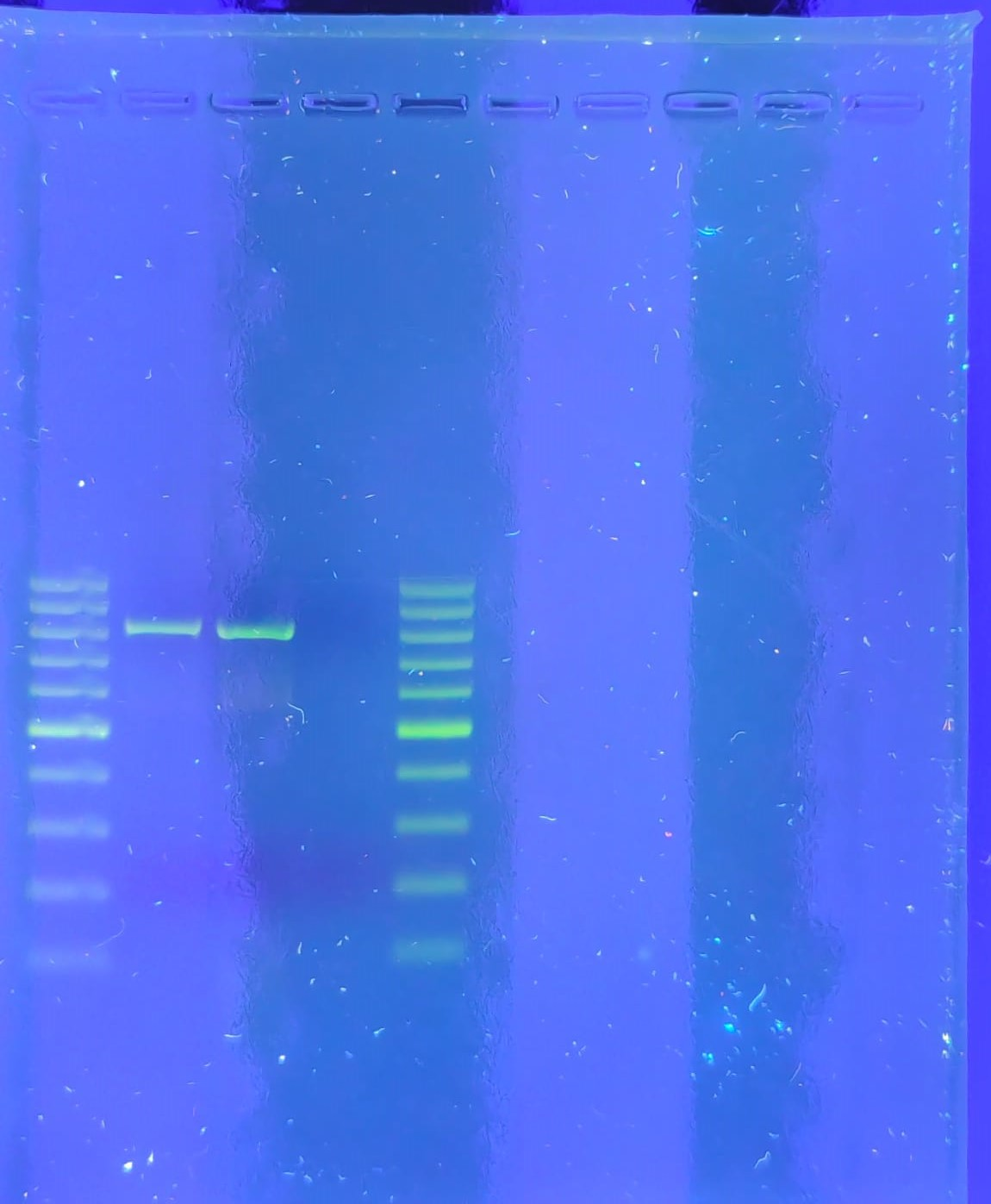

Supplement: Supplementary file 2 [file Image2.tiff]
